# A novel medication decision gene signature predicts response to individualized therapy and prognosis outcomes in hepatocellular carcinoma patients

**DOI:** 10.3389/fimmu.2022.990571

**Published:** 2022-10-07

**Authors:** Jingsheng Yuan, Zijian Liu, Zhenru Wu, Lvnan Yan, Jiayin Yang, Yujun Shi

**Affiliations:** ^1^ Department of Liver Surgery and Liver Transplantation Center, West China Hospital of Sichuan University, Chengdu, China; ^2^ Laboratory of Liver Transplantation, Frontiers Science Center for Disease-related Molecular Network, West China Hospital of Sichuan University, Chengdu, China; ^3^ Department of Head and Neck Oncology, Cancer Center and State Key Laboratory of Biotherapy, West China Hospital of Sichuan University, Chengdu, China; ^4^ Department of Radiation Oncology, Cancer Center and State Key Laboratory of Biotherapy, West China Hospital of Sichuan University, Chengdu, China; ^5^ Laboratory of Pathology, Key Laboratory of Transplant Engineering and Immunology, NHC, West China Hospital of Sichuan University, Chengdu, China

**Keywords:** hepatocellular carcinoma, immunotherapy, multikinase inhibitor, individualized therapy, prognostic model

## Abstract

Molecular targeted therapy has shown potential in hepatocellular carcinoma (HCC) patients, and immunotherapy applications are developing rapidly. However, clinical guidance for making individualized therapy decisions for HCC patients remains lacking. MDH (Medication Decision in HCC) gene signatures comprising 70 genes were screened using transcriptomic data from multikinase inhibitor (TKI)-resistant HCC cells and HCC patient-derived xenograft model (PDX) models. Four MDH subtypes with distinct biological and clinical characteristics were defined by unsupervised cluster analysis of HCC data from The Cancer Genome Atlas (TCGA) database. To facilitate individualized and reasonable clinical guidance for each HCC patient, we constructed the MDH score. Comprehensive analysis suggested high MDH scores were associated with TKI resistance, a high proportion of stromal cell infiltration and poor survival outcomes. We recommend concomitant stromal activity intervention and immunotherapy for this type of HCC. Moreover, low MDH scores indicate TKI sensitivity, and a combination of targeted and immunotherapy is recommended. The nomogram constructed by iteration least absolute shrinkage and selection operator (LASSO) Cox regression analysis successfully predicted 3- or 5-year survival outcomes and mortality risks of HCC patients. In conclusion, TKI resistance model-based MDH gene signatures provide novel insight into potential mechanisms of drug resistance and heterogeneity in HCC. Integrative analysis plus a simplified decision model may aid personalized treatment and prognostic assessment among HCC patients.

## Introduction

Hepatocellular carcinoma (HCC) is one of the most common cancers worldwide and the third leading cause of cancer-related deaths (1). HCC represents ~90% of all primary liver cancers (2). Approximately half of HCC patients eventually receive systemic therapy during their disease course, especially in the advanced stages of the disease (3). The number of available systemic treatment drugs for HCC is gradually increasing (4). Sorafenib was the first multikinase inhibitor (TKI) for HCC to be approved by the FDA and increased the median patient overall survival time from 8 to 11 months (5). Several newer TKIs, including first-line levatinib and second-line regorafenib and cabozantinib, have gradually been incorporated into clinical approaches (6). Additionally, immune checkpoint inhibitors have been an option for HCC treatment since 2020, and the combination of targeted therapies and immunotherapy is emerging as the most promising clinical treatment option for this disease (7). Notably, studies have revealed that patients receiving individualized therapy have better clinical outcomes (8). However, there are no appropriate clinical recommendations for precise drug selection in HCC patients. Furthermore, despite the clinical benefits of systemic therapy, the improvement in patient prognosis is limited and has occurred gradually. Therefore, further studies are necessary to investigate the underlying mechanisms of drug resistance in HCC and facilitate accurate medication decision-making for HCC patients.

Most of the current clinical treatments for tumours address specific genes or genomes, but researchers realize that these specific genes or genomes comprise a limited number of targets. In addition to the already widely applied analysis of carcinogenic signalling pathways, previous studies have proposed tumour mutation burden (TMB) to measure gene mutation frequency (9), the mRNA-based stemness index (mRNAsi) to evaluate the stemness of tumours (10), and the ferroptosis potential index (FPI) to assess the vulnerability of tumour cells to ferroptosis (11). Moreover, tumour immune cell markers for immune infiltration analysis and the Estimation of Stromal and Immune cells in malignant tumours using Expression data (ESTIMATE) algorithm for calculating stromal and immune cell proportions have recently been proposed (12, 13). Tumour genetic alterations are complex, and these genomes comprise only a small fraction of oncogenic transformation; thus, it is necessary to identify new biomarkers or tumour-associated genomic profiles. Whole transcriptome sequencing offers new opportunities to dissect tumour heterogeneity and complexity, providing a multifaceted view of tumour characteristics for exploring and developing new therapeutic strategies, potentially driving further identification and optimization of individualized treatment options for cancer patients (14).

Although many models for tumour assessment or classification have been proposed using transcriptome sequencing or multiomics studies, most of these models are based on a known mutated gene, tumour phenotype or gene cluster (15), and few have been constructed starting from a tumour drug resistance-associated gene cluster. Multidrug resistance (MDR) refers to drug resistance to several antitumour drugs with diverse structures and various mechanisms after tumours develop resistance to certain chemotherapeutic drugs (16), which is the main reason for the failure of chemotherapy. Interestingly, our study also observed this MDR phenomenon in HCC against TKIs. Given this mechanism of MDR, we speculate that there may be a relevant set of genes mediating MDR in HCC TKI therapy. Thus, our study proposed the MDH (Medication Decision in HCC) gene signatures and characterized the MDH subtype of HCC. Moreover, we further performed MDH scoring to predict individualized therapy and prognosis outcomes in HCC patients.

## Materials and methods

### Clinical samples

A retrospective analysis of HCC resection samples from the West China Hospital of Sichuan University from May 2019 to December 2020 was performed. HCC samples taken at the initial surgery that met the following criteria were included: 1. a history of sorafenib; 2. prognosis information after sorafenib therapy was available; 3. a confirmed clinicopathological diagnosis of HCC through pathology reports. Formalin-fixed and paraffin-embedded HCC tumour specimens were obtained from a tissue bank maintained at the West China Hospital. According to the Modified Response Evaluation Criteria in Solid Tumours (mRECIST) (17), we obtained 20 HCC samples from patients with progressive disease (PD) and 20 HCC samples from patients with partial response (PR). PD patient samples were considered sorafenib-resistant. The PR patient samples were considered sorafenib-sensitive. This clinical sample study was approved by the Ethics Committee on Biomedical Research, West China Hospital of Sichuan University (2016, no. 120). Informed consent was obtained from all patients or their relatives. The details of HCC patients are listed in **Table S1**.

### Cell culture

Huh7 and HepG2 cell lines were purchased from the National Collection of Authenticated Cell Cultures (Shanghai, China) and were cultured in complete medium containing Dulbecco’s modified Eagle’s medium (DMEM) (HyClone, UT, USA) supplemented with 10% foetal bovine serum (FBS) (Gibco, NY, USA), 1000 U/ml penicillin and 100 μg/ml streptomycin (HyClone, UT, USA) and were grown in a humidified air atmosphere containing 5% CO_2_ at 37°C. All cell lines were analysed by STR profiling for cell line authentication, and routine mycoplasma detection was performed. Sorafenib-resistant HCC cell lines generated from Huh7 and HepG2 parental cells were cultured as previously described (18).

### Patient-derived xenograft model

A patient-derived xenograft model (PDX) was established as previously reported (19). Briefly, freshly procured hepatocellular carcinoma samples were cut into small tissue blocks (~50 mm^3^) and kept in tissue culture media on ice until use (<5 hours). Six-week-old nude mice were anaesthetized with an isoflurane/oxygen mixture. Engrafted tissue blocks were carefully sealed under the skin of the mice using a tissue adhesive (Vetbond). Dosing was initiated when tumours reached approximately 0.2 cm^3^, and sorafenib (10 mg/kg) was administered thereafter every day. Tumour that regressed significantly were considered sorafenib-sensitive HCC. Tumours did not regress significantly despite continuous dosing were considered sorafenib-induced drug-resistant HCC. Human samples were obtained patient consent and approval from the institutional review board, conforming to the ethical guidelines of the 1975 Declaration of Helsinki. Animals received humane care, and the Institutional Animal Care and Use Committee (IACUC) approved all animal experiments (2020351A). Subsequently, tumour samples were collected by GeneChem Co., Ltd. (Shanghai, China) for transcriptome sequencing and subsequent data analysis. Three PDX samples per group were used for sequencing.

### Transcriptome sequencing of HCC cells

Total RNA from HCC cells was processed and extracted using a TRIzol reagent kit (Takara, Dalian, China) and collected by Novogene Co., Ltd. (Tianjin, China) for transcriptome sequencing and subsequent data analysis. Three samples of the parental and sorafenib-resistant HCC cells were used for sequencing.

### Cell counting kit-8

The cell counting kit-8 (CCK-8) was performed as previously described (20).

### Clonogenic cell survival assay

The indicated cells were treated with sorafenib (Selleck, S7397, TX, USA) and lenvatinib (Selleck, S1164) for 24 h, and 3000 cells were plated into 6-well plates. Two weeks later, the colonies were fixed with 4% paraformaldehyde, followed by 30 min of incubation with 0.1% crystal violet. The 6-well plates were washed and then visualized.

### Immunohistochemistry

Immunohistochemistry (IHC) was performed as previously described (18). The primary antibodies used in this study are listed in **Table S2**.

### Data sources and preprocessing

The raw data of fragment per kilobase (FPKM) values and liver hepatocellular carcinoma (LIHC) clinical information in The Cancer Genome Atlas (TCGA) and International Cancer Genome Consortium (ICGC) datasets were downloaded from the UCSC XENA database. The series matrix files of the Affymetrix and Illumina-generated microarray for GSE109211 and GSE73571 were directly downloaded from the Gene Expression Omnibus (GEO) database. The immunotherapy cohort of patients with metastatic urothelial carcinoma treated with the anti-PD-L1 antibody atezolizumab (IMvigor210) was obtained according to official guidelines. All the information about the public datasets is summarized in **Table S3**.

### Functional and pathway enrichment analysis

The Kyoto Encyclopedia of Genes and Genomes (KEGG) pathway analysis results were obtained from The Database for Annotation, Visualization and Integrated Discovery (DAVID) v6.8 (21). Gene set enrichment analysis (GSEA) was performed on the expression data of a specified set of transcripts according to previously published expression methods (22). To further estimate pathway and biological process activity variations in samples from expression datasets, we performed Gene Set Variation Analysis (GSVA) enrichment analysis using the “GSVA” R package, a nonparametric and unsupervised method (23). The gene set “c5.all.v6.2. symbols” was downloaded from the MSigDB database on the GSEA website, and another published pathway gene set is summarized in **Table S4** (24).

### Assessment of the tumour immune microenvironment

As described above, we used the GSVA method to perform gene-set enrichment analysis to quantify the relative abundance of each infiltrating cell in a single sample. The immune cell markers used in this study were extracted from two previously published authoritative studies (12, 25), as shown in **Table S5, S6**, referred to as immune cell signatures 1 and 2, respectively. Additionally, ESTIMATE was used to infer the fraction of stromal and immune cells in the samples (13).

### Unsupervised clustering for MDH gene signatures

Based on the expression of the MDH gene signatures, unsupervised clustering analysis was performed using the TCGA-LIHC dataset to identify distinct MDH gene expression patterns and to classify HCC patients for further analysis. The number of clusters and their stability were determined by a consensus clustering algorithm. We performed the above steps using the “ConsensuClusterPlus” R package and performed 1000 repetitions to guarantee the stability of the classification.

### Dimension reduction and generation of the MDH score

To quantify the MDH expression patterns of individual tumours, we constructed a scoring system based on the principal component analysis (PCA) score method to evaluate the MDA score of individual HCC patients. Gene patterns were annotated using the clusterProfiler R package. A consensus clustering algorithm was applied to define gene clusters, and PCA was performed (26). Principal components 1 and 2 were both used as gene feature scores. After obtaining the prognostic value of each gene signature score, we applied a method similar to gene expression grade index (GGI) to define the MDH score for each patient:


MDHscore=Σ(PC1i+PC2i)

where i is the expression of MDH phenotype-related genes.

### Calculation of the ferroptosis potential index (FPI) and mRNA-based stemness index (mRNAsi)

The index representing ferroptosis susceptibility was established from the expression data of ferroptosis core machine genes, including positive components and negative components, as shown in **Table S7**. The enrichment score (ES) of the gene set was calculated using the ‘GSVA’ R package, and the FPI was calculated as follows (11):


FPI=ES(positive)-ES(negative)

To assess the stemness of cancer cells, a one-class logistic regression algorithm, mRNAsi, was used to calculate the stemness index for each HCC sample using the workflow available on a previously established database (10).

### Predicting response to immunotherapy

The Tumor Immune Dysfunction and Exclusion (TIDE) algorithm was used to predict HCC responsiveness to immunotherapy (27). The TIDE algorithm captures two different mechanisms of the tumour immune escape score, including immunosuppressive factor rejection of tumour-infiltrating cytotoxic T lymphocytes (CTLs) score (exclusion) and CTLs dysfunction score (dysfunction). Additionally, the TIDE algorithm obtains three cell types that limit T-cell infiltration into tumours, myeloid-derived suppressor cells (MDSCs), cancer-associated fibroblasts (CAFs), and the M2 subtype of tumour-associated macrophages (TAM-M2s).

### Establishment of the MDH Risk score

Univariate Cox regression analysis was used to observe the correlation between the expression level of the MDH gene set in the TCGA and patient prognosis. Genes with p< 0.01 in univariate Cox regression analysis were included in the construction of prognostic risk models. Next, the iteration least absolute shrinkage and selection operator (LASSO) Cox regression model was used to screen for the best gene signature involved in patient resistance to TKIs (28). Finally, multivariate Cox proportional hazards regression was performed to model the risk score and previously published algorithms were used to calculate the risk score. The median of risk scores was used as a cut-off to stratify patients into high- and low-risk groups. Differences in survival between the high- and low-risk groups were further compared using Kaplan–Meier analysis. Univariate Cox regression analysis and multivariate Cox regression analysis were performed using the “survminer” package in R to investigate the relationship between risk score and prognosis. Using the “timeROC” package in R, the area under the curve (AUC) was used to test the performance of the classifier. We also assessed the prognostic value of these genes in the model.

### Development and validation of the prognostic nomogram

Based on the risk scores of clinical risk factors and multivariate Cox regression coefficients, a prognostic nomogram was built using the “rms” R package, and the predictive accuracy of this nomogram was assessed using the calibration curve and the concordance index.

### Statistical Analysis

All statistical calculations were performed using R software (version 3.6.1). Analysis of differentially expressed genes between different defined groups was performed using the empirical Bayesian approach of the “limma” R package, with significance criteria set as adjusted P-value< 0.05 and log2 |fold change (FC)| > 1. Differentially expressed mRNAs were visualized as heatmaps and volcano plots in R using the packages “pheatmap” and “ggplot2”. To calculate the TMB per megabase, the total number of mutations counted was divided by the size of the coding region of the targeted territory in the TCGA-LIHC cohort (9). The mutation landscape oncoprint was generated using the R package “ComplexHeatmap. The comparison of normally distributed variables between the two groups was performed using an unpaired t test, and the statistical significance of the nonnormally distributed variables was estimated using the Mann–Whitney U test (Wilcoxon rank-sum test). Spearman’s correlation analysis was performed to calculate the correlation coefficient between the two factors. Based on the correlation between gene expression and patient survival, the optimal cut-off point for each dataset was determined using the “survminer” R package, and the “surv-cutpoint” function was used to repeat all potential cut-off points to obtain the maximum rank statistic, divided into two groups: high and low. Survival curves for prognostic analysis were generated using the Kaplan–Meier method, and significant differences were determined using the log-rank test. The false discovery rate (FDR) method was used to adjust the P-value for multiple comparisons, and statistical significance was set at p<0.05; that is, the FDR was less than 0.05. The asterisks represent the statistical P-value (*P< 0.05; **P< 0.01; ***P< 0.001).

## Results

### Establishment of MDH gene signatures based on TKI-resistant HCC

**Figure 1:**
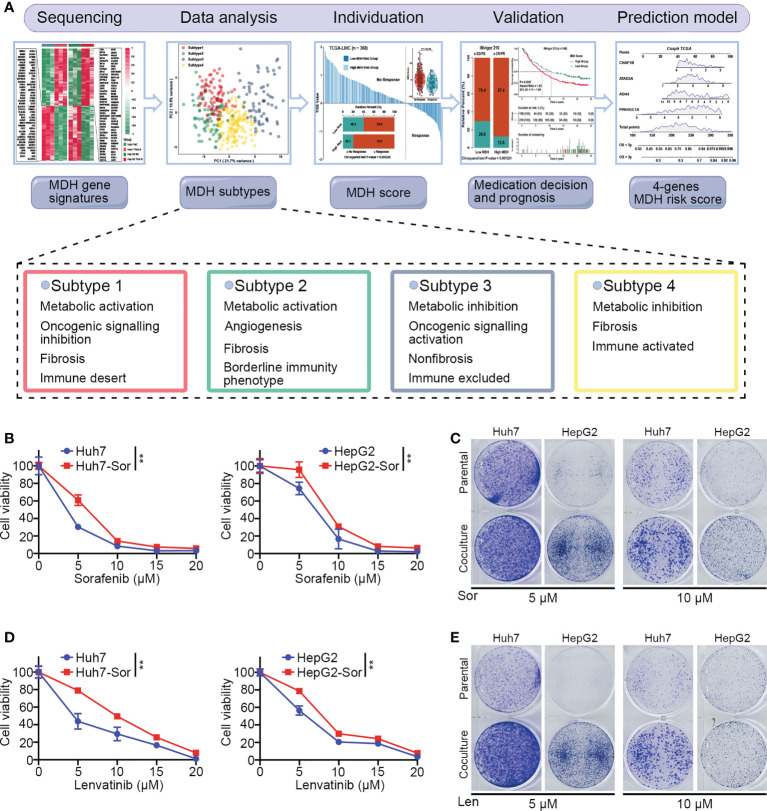
The culture of HCC sorafenib-resistant cell lines. **(A)** Schematic overview of the workflow in this study. **(B)** CCK-8 assays for parental HCC cells and corresponding sorafenib-resistant HCC cells treated with a range of concentrations of sorafenib. Cell viability was assessed 3 d after sorafenib treatment. **(C)** Clonogenic cell survival assay in specified cells treated with sorafenib for 24 h. **(D)** CCK-8 assays for parental HCC cells and corresponding sorafenib-resistant HCC cells treated with a range of concentrations of lenvatinib. Cell viability was assessed 5 d after lenvatinib treatment. **(E)** Clonogenic cell survival assay in specified cells treated with 10 μmol lenvatinib for 24 h. The asterisks in B and D represent the statistical p-value (**P< 0.01).

**Figure 2:**
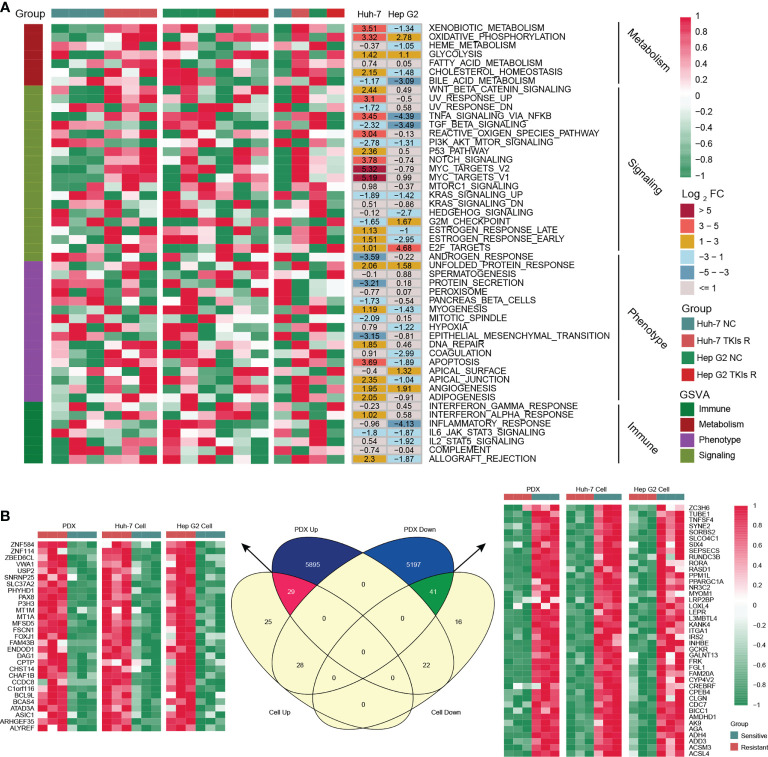
Generation of the MDH gene signatures. **(A)** GSEA enrichment analysis of differentially expressed genes (DEGs) between parental HCC cells and corresponding sorafenib-resistant HCC cells. **(B)** Venn diagram of MDH gene signatures acquired from the sorafenib-resistant PDX model and HCC cell lines.

As shown in **Figure 1A**, we designed this study to construct MDH gene signatures and investigate their potential value for individualized therapy and clinical application in HCC. We first cultured sorafenib-resistant HCC cell lines using our previously published method (**Figures 1B, C**) (18). Interestingly, CCK-8 and clonogenic cell survival assays indicated that sorafenib-resistant HCC cells were also significantly less sensitive to lenvatinib (**Figures 1D, E**), implying multidrug resistance. To delineate the alterations in gene expression and functional characteristics of TKI-resistant HCC cells, we conducted transcriptome sequencing of TKI-resistant HCC cells and parental HCC cells and further performed KEGG pathway enrichment analysis on the differentially expressed genes (DEGs). The results suggested that upregulated genes in TKI-resistant HCC cells were mainly enriched in the cell cycle and metabolic pathways (**Figure S1A**), while downregulated genes were mainly enriched in the FoxO signalling pathway, adherens junction and AMPK signalling pathway (**Figure S1B**). We further verified the above results using GSVA, which indicated four major variations in tumour metabolism, tumour-associated signalling, tumour phenotype and tumour immunity in TKI-resistant HCC cells (**Figure 2A**). Notably, tumour metabolism, such as oxidative phosphorylation, gluconeogenesis, and fatty acid metabolism, was dramatically enriched in TKI-resistant HCC cells. Among tumour-associated signalling and tumour phenotypes, TGF-β signalling, PI3K/AKT/mTOR signalling, KRAS signalling, and EMT signalling were remarkably enriched in parental HCC cells, while Wnt/β-catenin signalling, DNA repair and angiogenesis were significantly enriched in TKI-resistant HCC cells. Moreover, tumour immunity, such as the inflammatory response, IL6/JAK/STAT3 signalling and IL2/STAT5 signalling, was prominently enriched in parental HCC cells (**Figure 2A**). Finally, we obtained 161 genes from TKI-resistant HCC cells to establish MDH gene signatures (**Figures S1C, D**).

To further screen potential key genes *in vivo* for constructing MDH gene signatures, we developed and induced a TKI-resistant PDX model of HCC by *in vivo* sorafenib injection. Importantly, consistent with the *in vitro* analysis, our transcriptome sequencing and GSEA analysis demonstrated that metabolism-related signalling pathways were also markedly enriched in the TKI-resistant PDX model (**Figure S1E**). GSVA further emphasized that metabolism-related signalling is the main enriched pathway in TKI-resistant HCC, while EMT signalling, the inflammatory response and IL2/STAT5 signalling are the critical characteristic pathways in TKI-sensitive HCC (**Figure S1F**). These results revealed that among the myriad of signalling pathways, alterations in metabolism-related signalling, tumour phenotype and tumour immunity were potentially crucial mechanisms of TKI resistance in HCC. Combined with previous *in vitro* results, we further screened 70 genes to construct MDH gene signatures (**Figure 2B** and **Table S8**).

### Unsupervised analysis of MDH gene signatures revealed four HCC subtypes

**Figure 3:**
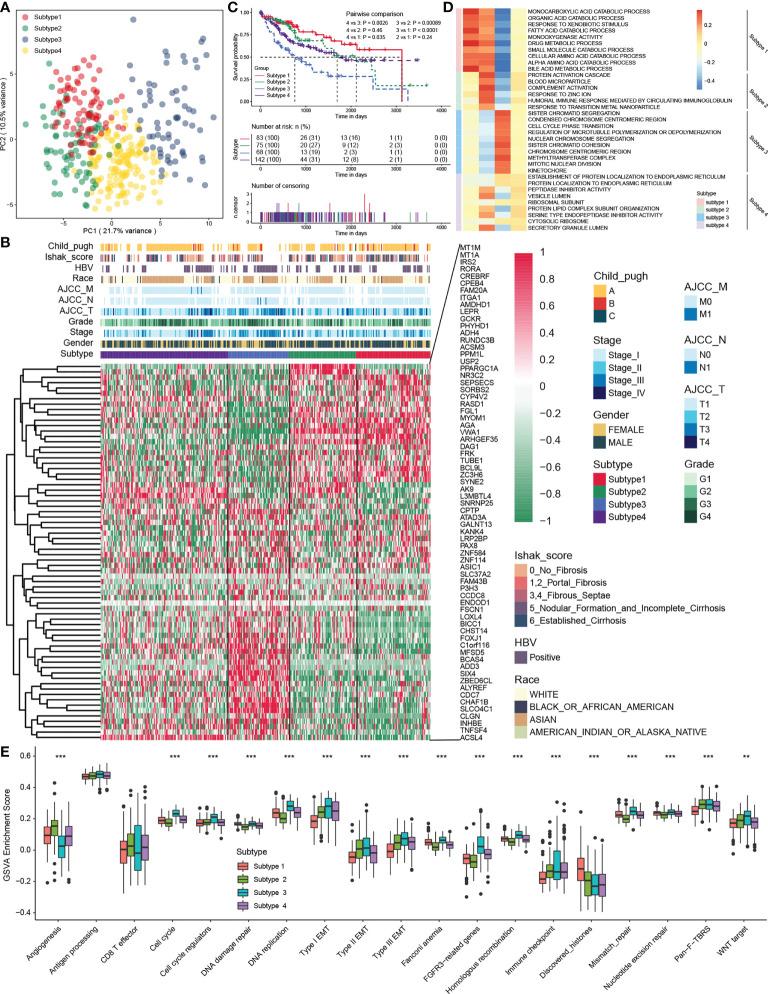
The four distinct MDH subtypes identified in HCC. **(A)** Principal component analysis for the transcriptome profiles in distinct MDH subtypes of the TCGA-LIHC cohort. **(B)** Unsupervised clustering of MDH gene signatures in the TCGA-LIHC cohort. **(C)** Survival analyses for distinct MDH subtypes of the TCGA-LIHC cohort. **(D)** GSVA enrichment analysis showing the activation states of biological pathways in distinct MDH subtypes of the TCGA-LIHC cohort. A heatmap was used to visualize these biological processes. **(E)** Differences in the expression of known signatures, including stromal activation-related signatures, tumour promotion-related signatures and immune activation-related signatures, in distinct MDH subtypes of the TCGA-LIHC cohort. The upper and lower ends of the boxes represent the interquartile range of values. The lines in the boxes represent the median value, and black dots show outliers. The asterisks represent the statistical p-value (**P< 0.01 and ***P< 0.001).

An unsupervised cluster analysis was performed using 368 HCC samples from the TCGA-LIHC dataset to recognize distinct HCC subtypes and investigate the potential value of screened MDH gene signatures in HCC. The consensus clustering algorithm determined the number and stability of clusters. This analysis revealed that the molecular profiles of HCC could be clustered into four distinct MDH subtypes (**Figure S2A**). PCA analysis further revealed significant distinctions in the transcriptional profiles among the four subtypes (**Figure 3A**). A combined heatmap was plotted to visualize MDH subtype gene expression levels and clinicopathological features to investigate the correlation between these subtypes and features in HCC (**Figure 3B**). Notably, we observed remarkable disparities in gene expression abundance among different MDH subtypes. Moreover, subtypes 3/4 were markedly associated with higher histological grade and more advanced staging in HCC, and subtype 3 was strongly associated with HBV infection. Subtypes 1/2/4 were all significantly associated with HCC fibrosis relative to subtype 3 (**Figure 3B**). Prognostic analysis of pairwise comparisons further indicated that patients with subtype 3 HCC had significantly worse overall survival rates than those with subtypes 1/2/4, while patients with subtype 1 had a relatively better prognosis than those with subtypes 3/4 (**Figure 3C**).

KEGG pathway enrichment analysis was performed utilizing the gene expression profiles of each MDH subtype from the TCGA-LIHC dataset to investigate the potential mechanisms contributing to the differences in clinical features and prognosis across MDH subtypes. Interestingly, the upregulated genes in subtype 1 were dramatically associated with tumour metabolism, including metabolic pathways and drug metabolism (**Figure S2B**), while tumour-associated signalling pathways, such as ECM receptor interaction, focal adhesion, the NFKB signalling pathway and the TNF signalling pathway, were markedly downregulated (**Figure S2C**). Conversely, the upregulated genes in subtype 3 were dramatically involved in tumour-associated signalling pathways, including the cell cycle, ECM receptor interaction, P53 signalling pathways, pathways in cancer, PI3K/AKT signalling pathways, focal adhesion and DNA replication (**Figure S2B**), while tumour metabolism in subtype 3 was remarkably suppressed (**Figure S2C**). Additionally, upregulated genes in subtype 2 and downregulated genes in subtype 1 were markedly enriched in tumour metabolism-related signalling, but genes in subtype 1/2 were not distinctly connected to oncogenic-associated signalling pathways (**Figures S2B, C**). We further presented a combined heatmap using GSVA to visualize the pathway differences between subtypes and the associations with clinical features of HCC, which also validated that metabolism-related signalling pathways were enriched in subtype 1, while oncogenic signalling pathways such as cell cycle and DNA replication were enriched in subtype 3 (**Figure 3D** and **Figure S3A**). Notably, GSVA enrichment analysis of the indicated pathway sets revealed that subtype 3 not only had the most remarkable correlation with signalling pathways such as the cell cycle, DNA replication, and EMT signalling but also had a strong association with tumour immune checkpoints, while subtype 2 was significantly correlated with angiogenesis (**Figure 3E**). In brief, our analysis revealed that HCC could be clustered into four distinct subtypes as shown in **Figure 1A**, namely, subtype 1, metabolic activation, oncogenic signalling inhibition, and fibrosis; subtype 2, metabolic activation, angiogenesis, and fibrosis; subtype 3, oncogenic signalling activation, metabolic inhibition, and nonfibrosis; and subtype 4, metabolic inhibition and fibrosis.

### Immune microenvironmental characteristics of four MDH subtypes in HCC

**Figure 4:**
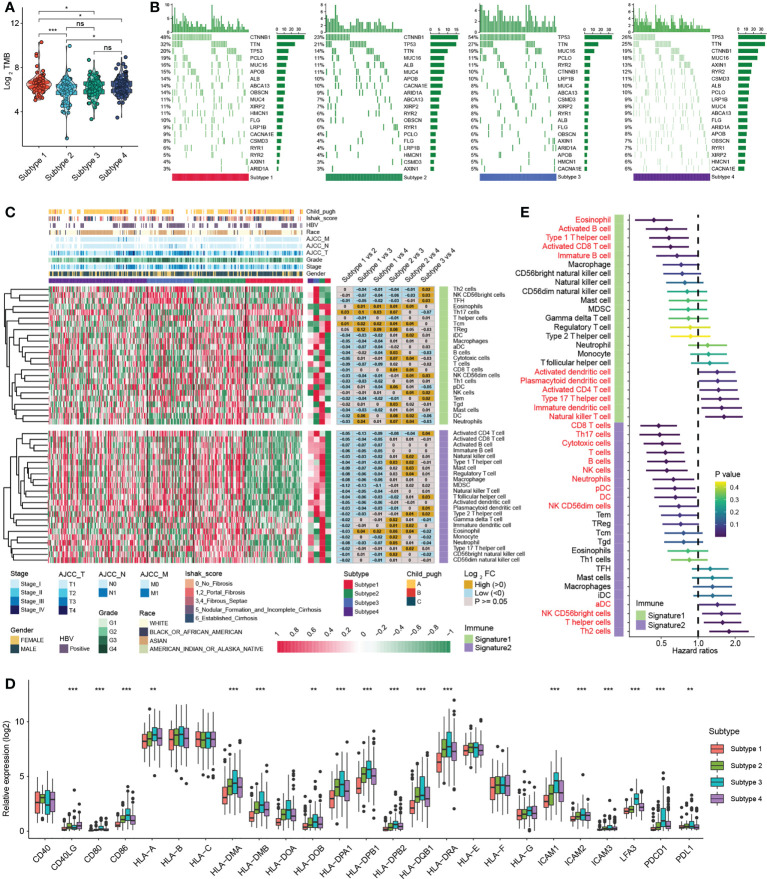
Immune infiltration analysis of distinct MDH subtypes in the TCGA-LIHC cohort. **(A)** Tumour mutation burden (TMB) in distinct MDH subtypes of the TCGA-LIHC cohort. **(B)** The gene mutation frequency in distinct MDH subtypes of the TCGA-LIHC cohort. Each column represents individual patients. The upper bar plot shows TMB. The number on the right indicates the mutation frequency in each gene. The right bar plot shows the proportion of each variant type. **(C)** Unsupervised clustering of two previously published immune cell gene signatures in distinct MDH subtypes of the TCGA-LIHC cohort. **(D)** Differences in the expression of MHC molecules, costimulatory molecules and adhesion molecules in distinct MDH subtypes of the TCGA-LIHC cohort. The upper and lower ends of the boxes represent the interquartile range of values. The lines in the boxes represent the median value, and black dots show outliers. The asterisks represent the statistical p-value (**P< 0.01 and ***P< 0.001). **(E)** Survival analyses for two previously published immune cell gene signatures based on the TCGA-LIHC cohort.

Significant progress has been recently achieved by combining immunotherapy and TKIs to treat HCC (29), illustrating the importance of the immune microenvironment in HCC therapy. Studies have revealed that tumours with a higher TMB are more responsive to immunotherapy (30). Our results suggested that the overall TMB of subtype 1 was markedly higher than that of other subtypes with mutations mainly derived from CTNNB1 gene (**Figures 4A, B**), while the mutation of P53 gene in subtype 3 was remarkably more frequent than that of other subtypes (**Figure 4B**). Next, we analysed the association of each subtype with the tumour immune microenvironment. Based on the ESTIMATE algorithm, subtype 1 had the poorest immune cell infiltration and stromal cell proportions and had the worst ESTIMATE score relative to other subtypes. Moreover, there were no statistical differences in immune cell infiltration between subtypes 2, 3 and 4, whereas subtype 2 had comparatively higher levels of stromal cell infiltration and ESTIMATE scores (**Figure S4A**). According to previously published methods (12, 25), we further calculated the types of infiltrating immune cells for each subtype and performed pairwise comparisons to further elucidate the differences in immune cells between subtypes. Consistently, the analysis indicated that subtype 1 had an extremely poor innate immune cell infiltration compared to subtypes 2/3/4, including T cells, B cells, natural killer cells, macrophages, eosinophils, mast cells, monocytes, MDSCs, and plasmacytoid dendritic cells (**Figure 4C**). Most solid tumours exhibit three main immunological phenotypes, termed immune inflamed, immune excluded and immune desert (31). Thus, MDH subtype 1 in HCC was classified as an immune desert. Indeed, an overall decrease in the expression of MHC molecules, adhesion molecules and immune checkpoints in subtype 1 was further confirmed (**Figure 4D**).

In contrast, natural killer CD56^+^ cells, Th2 cells, plasmacytoid dendritic cells (DCs) and activated CD4 T immune cells were abundant in subtype 3 (**Figure 4C**). Our prognostic analysis implied that natural killer CD56^+^ cells, Th2 cells, plasmacytoid DCs and activated CD4 T immune cell infiltration were negative factors for HCC patients (**Figure 4E**). Notably, MHC molecules and adhesion molecules were more abundant in HCC subtype 3 than in subtypes 1/2/4 (**Figure 4D**). Moreover, PD1 and PD-L1 expression levels were dramatically increased in subtype 3. Cytokines with analogous expression trends include CD80 and CD86, ligands for CTLA-4 (**Figure 4D**). In theory, subtype 3 should be the tumour type with a better immune response in HCC; however, patients with subtype 3 did not display a matching survival advantage (**Figure 3C**). Previous studies have identified that tumours with an immune-excluded phenotype also exhibit infiltration of abundant immune cells, whereas these immune cells are retained in the stroma surrounding tumour cell nests rather than penetrating the parenchyma (31). Stromal activation is an essential driver of T-cell suppression (32). Our ESTIMATE analysis indicated that subtype 3 was markedly associated with stromal cell infiltration (**Figure S4A**). Our GSVA further implied that subtype 3 was associated with dramatically enhanced stromal activation, including increased levels of TGF-β signalling and EMT signalling (**Figure S3A**). Thus, MDH subtype 3 in HCC was classified as immune-excluded.

Furthermore, subtype 2 and subtype 4 contained abundant activated CD8 T cells, activated B cells, immature B cells and eosinophil cells, and HCC subtype 2 contained abundant DCs, including activated DCs (aDC), immature DCs (iDC), and plasmacytoid DCs (pDC) (**Figure 4C**). Our prognostic analysis implied that activated CD8 T cells, activated B cells and immature B cells, eosinophils and plasmacytoid DC infiltration were protective factors in HCC, while activated DCs and immature DCs were detrimental factors (**Figure 4E**). DCs are responsible for antigen presentation and the activation of naive T cells, bridging innate and adaptive immunity, and their activation depends on the expression levels of MHC molecules, costimulatory molecules, and adhesion molecules (24). Our analysis indicated that the expression of MHC molecules, costimulatory molecules and adhesion molecules was indeed somewhat higher in subtype 2 than in subtypes 1/4 (**Figure 4D**). However, we noticed that subtype 2 HCC had more abundant stromal cell infiltration than subtype 1/4 (**Figure S4A**), and angiogenesis was also enriched in subtype 2 (**Figure 3E**), suggesting that stromal cells in subtype 2 HCC were also partially activated. Therefore, subtype 4 was classified as immune-activated HCC, while subtype 2 was classified as at the borderline between immune-activated and immune-excluded.

### The MDH score predicts the TKI response and prognosis in HCC

The ferroptosis index and mRNAsi are also scores used to measure tumour malignancy (10, 11). Notably, the mRNAsi score and ferroptosis index of HCC were significantly higher than those of nontumor liver tissues, indicating that tumour stemness and ferroptosis susceptibility in HCC were markedly higher than those of nontumor tissue (**Figures S5A, B**). Notably, subtype 3 had a higher ferroptosis index than subtypes 1/2/4 (**Figure S5A**), but the mRNAsi across subtypes could not be completely distinguished (**Figure S5B**). Combined with previous ESTIMATE scores (**Figure S4A**), these results indicated that although MDH gene signatures can classify HCC into four subtypes with individual features, there are restrictions in quantifying various known features in HCC. We speculated that each gene in the MDH gene signature has different weights in predicting malignancy and individuality characteristics in HCC. Additionally, the above analysis was only based on the HCC patient population and could not precisely forecast and quantitate the gene expression patterns of individual patients. Considering the individual heterogeneity and complexity of HCC, we constructed an MDH score based on the PCA-score algorithm to group each HCC patient and provide appropriate clinical guidance (33).

**Figure 5:**
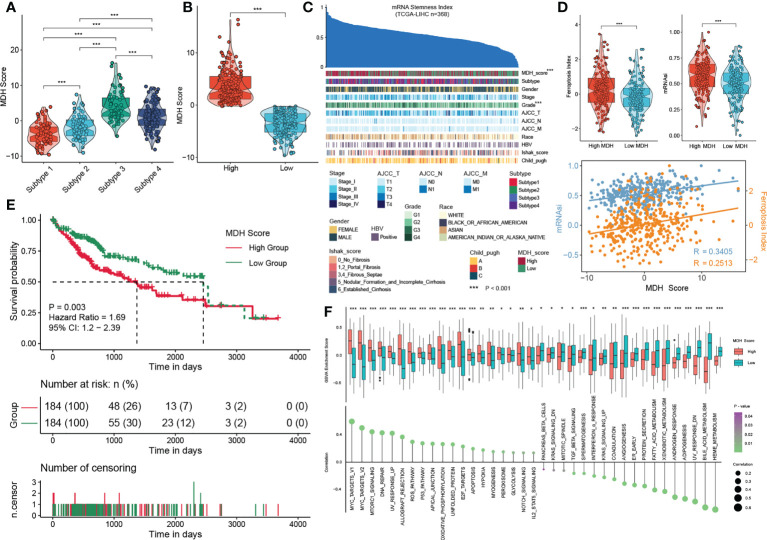
Construction of the MDH score. **(A)** MDH score of distinct MDH subtypes in the TCGA-LIHC cohort. **(B)** The TCGA-LIHC cohort was divided into two groups based on median expression values of MDH scores. **(C)** An overview of the association between known clinical features and mRNAsi in patients in the TCGA-LIHC cohort. Columns represent samples sorted by mRNAsi from low to high (top row). Rows represent known clinical and molecular features. **(D)** mRNAsi score and ferroptosis index in the MDH high- or low-score groups of the TCGA-LIHC cohort. Correlation analysis of mRNAsi scores, ferroptosis index and MDH score in patients in the TCGA-LIHC cohort. **(E)** Survival analyses for MDH high- or low-score groups of the TCGA-LIHC cohort. **(F)** GSVA enrichment analysis showing the activation states of biological pathways in the MDH high- or low-score groups. The upper and lower ends of the boxes represent the interquartile range of values. The lines in the boxes represent the median value, and black dots show outliers. The asterisks in A, B, D and F represent the statistical p-value (*P< 0.05; **P< 0.01 and ***P< 0.001).

We first confirmed that there were remarkable differences in MDH scores among each subtype: subtype 3> subtype 4> subtype 2> subtype 1 (**Figure 5A**). We then divided the HCC patients in the TCGA-LIHC database into two groups according to the MDH score to facilitate subsequent analysis (**Figure 5B**). The patients with high MDH scores were mainly derived from subtype 3/4 HCC, while those with low MDH scores mostly originated from subtype 1/2 HCC (**Figure S5C**). We found that a high MDH score in HCC was dramatically associated with a high ferroptosis index and mRNAsi score (**Figures 5C, D**), and the correlation analysis revealed that the MDH score was remarkably positively correlated with the ferroptosis index and mRNAsi score (**Figure 5D**). We calculated the MDH scores of established TKI-resistant HCC cell lines and HCC TKI-resistant PDX models to further validate our results. The results indicated a significant distinction in the gene transcription profiles between the MDH score groups. Moreover, MDH scores were considerably higher in TKI-resistant HCC than in TKI-sensitive HCC, and MDH scores were significantly positively associated with the ferroptosis index and mRNAsi score (**Figures S5D, S5E**). Consistently, datasets from the GEO database were utilized to confirm the potential value of MDH scores in discriminating gene expression profiles and predicting TKI responses, the ferroptosis index and the mRNAsi score (**Figures S5F, S5G**). These results revealed that higher MDH scores in HCC were associated with increased TKI resistance, higher tumour stemness and reduced ferroptosis susceptibility. More importantly, clinical analysis indicated that HCC with a high MDH score was dramatically correlated with a higher histological grade and Ishak score (**Figure S5C**). Moreover, HCC patients with high MDH scores had markedly worse overall survival outcomes than those with low MDH scores (**Figure 5E**). The GSVA further implied that P53 signalling, ROS signalling, oxidative phosphorylation, glycolysis, IL2/STAT5 signalling, and DNA repair were remarkably activated in HCC with high MDH scores, while KRAS signalling, angiogenesis, adipogenesis and fatty acid metabolism were inhibited (**Figure 5F**).

### The MDH score predicts the response to immunotherapy in HCC

**Figure 6:**
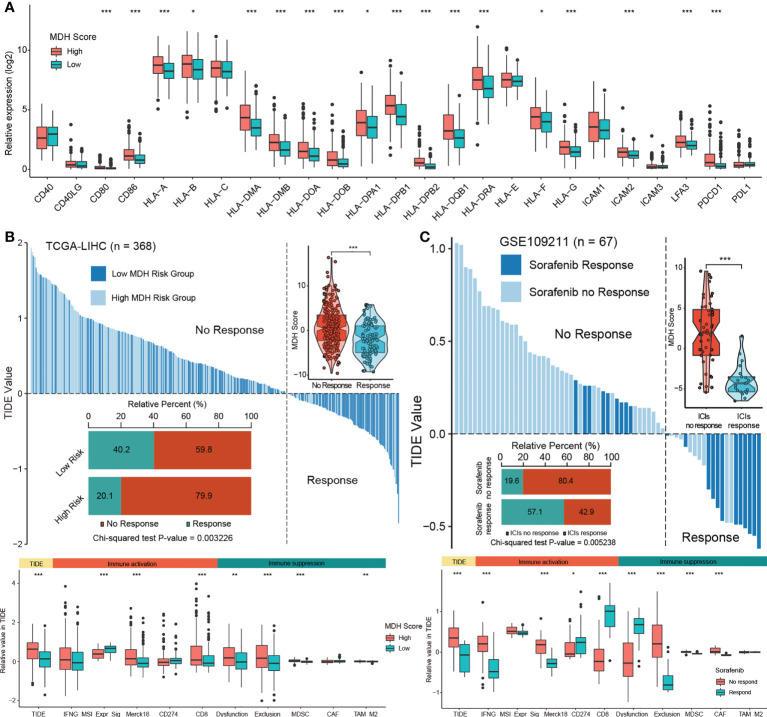
The MDH score predicts the immunotherapy response in HCC. **(A)** Differences in the expression of MHC molecules, costimulatory molecules and adhesion molecules in the MDH high- or low-score groups of the TCGA-LIHC cohort. The upper and lower ends of the boxes represent the interquartile range of values. The lines in the boxes represent the median value, and black dots show outliers. **(B)** TIDE value of MDH high- or low-score groups of TCGA-LIHC cohort. **(C)** TIDE values of sorafenib responders and nonresponders in the GSE109211 cohort. The chi-square test was used to calculate significant differences in **(B, C)** The asterisks represent the statistical p-value (*P< 0.05; **P< 0.01 and ***P< 0.001).

Next, we evaluated the relationship between MDH scores and immune cell infiltration using the ESTIMATE algorithm (13). No significant distinction was found in stromal cells between the two groups of HCC patients, but both immune cell infiltration and ESTIMATE scores were higher in HCC with high MDH scores than in HCC with low MDH scores (**Figure S6A**). Notably, MDH scores were markedly positively correlated with CD8 T cell, CD4 T cell, NK cell, B cell, DC and macrophage infiltration but significantly negatively correlated with eosinophil cell infiltration in HCC (**Figures S6B, C**). However, as previously stated, HCC patients with higher CD8 T cell, B cell and pDC infiltration had a better prognosis than HCC patients with lower CD8 T cell, B cell and pDC levels (**Figure 4E**), which appears to contradict the true overall survival time of HCC patients. This contradictory actual overall survival outcome implied that patients with high MDH scores may have an immune-excluded phenotype. Supporting insights also included that the expression levels of MHC molecules, costimulatory molecules, and adhesion molecules in high-MDH-score HCC patients were comprehensively elevated (**Figures 6A**, **S6D**), and the MDH score was positively correlated with the expression levels of PD1, CD80 and CD86 (**Figure 6A**).

To further evaluate whether MDH scores could predict immunotherapy response in HCC, we applied the TIDE algorithm to the TCGA-LIHC dataset. Studies have suggested that high TIDE scores are associated with poorer immune checkpoint inhibition treatment efficacy and worse overall survival outcomes in patients treated with anti-PD1 and anti-CTLA4 (27). Importantly, there is a strong positive correlation between MDH scores and TIDE scores (**Figure 6B**), indicating that HCC patients with high MDH scores respond poorly to immunotherapy. Moreover, our results suggested that MDH scores were positively correlated with CD8 and T cell inflamed (Merck18) levels and negatively correlated with microsatellite steady-state (MSI). We also observed that the MDH score was positively correlated with MDSC and TAM-M2 cell levels. Meanwhile, the MDH score was directly correlated with immune dysfunction and exclusion (**Figure 6B**), further supporting that HCC with a high MDH score is tightly associated with the immune-excluded phenotype. TIDE analysis of a testing dataset GSE109211 further demonstrated that HCC with a high MDH score, although responsive to sorafenib, was markedly less responsive to immunotherapy (**Figure 6C**). Correlation analysis also indicated that HCC with a high MDH score contained abundant immunosuppressive cells, including MDSCs and CAFs. Importantly, HCC with a high MDH score was indeed significantly positively correlated with immune dysfunction and exclusion (**Figure 6C**). These results revealed that HCC patients with a high MDH score had more immune cell infiltration than those with a low MDH score, but most of the cells were immune-escape and immunosuppressive cells; thus, HCC patients with a low MDH score responded better to immunotherapy.

### Validation of the MDH score for therapy selection in HCC

**Figure 7:**
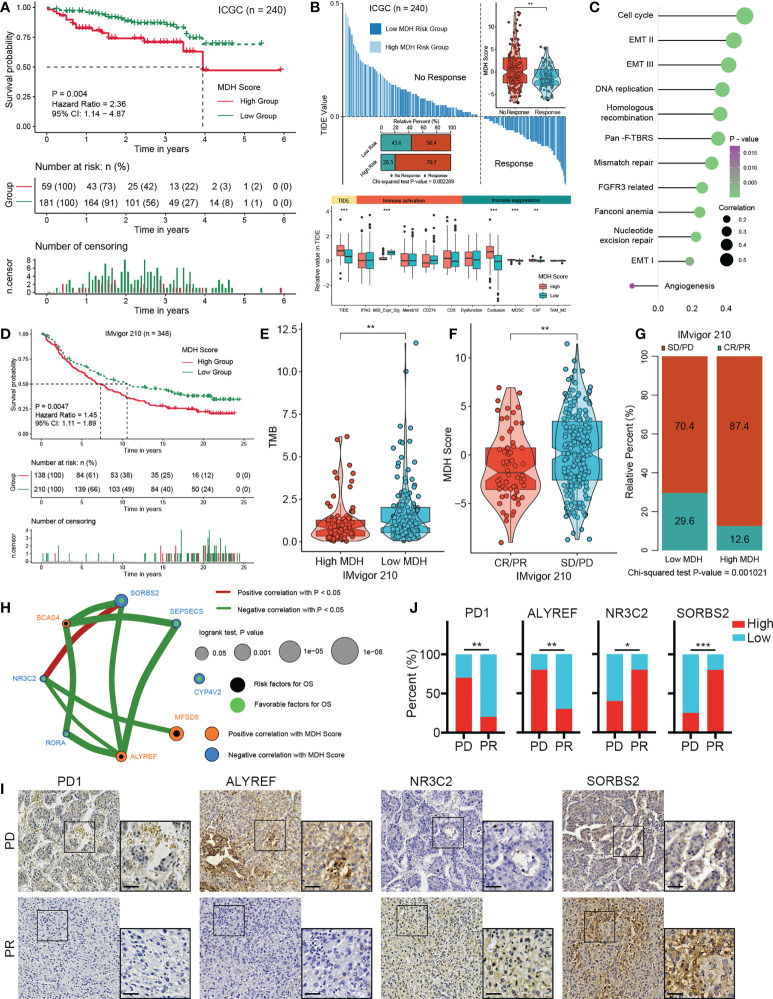
Validation of the MDH score for the immunotherapy response in HCC. **(A)** Survival analyses for MDH high- or low-score groups of the ICGC-LIHC cohort. **(B)** TIDE value of MDH high- or low-score groups in the ICGC-LIHC cohort. The chi-square test was used to calculate significant differences. **(C)** Differences expression of known signatures, including stromal activation-related signatures, tumour promotion-related signatures and immune activation-related signatures, in the MDH high- or low-score groups of the ICGC-LIHC cohort. **(D)** Survival analyses for MDH high- or low-score groups of the IMvigor210 cohort. **(E)** TMB score in the MDH high- or low-score groups of the IMvigor210 cohort. **(F)** MDH score in complete response/partial response (CR/PR) patients and stable disease/progressive disease (SD/PD) patients in the IMvigor210 cohort. **(G)** Correlation analysis of the MDH score with TKI response in the IMvigor210 cohort. The chi-square test was used to calculate significant differences. **(H)** The interaction between 8 hub genes in the MDH gene signatures of the TCGA-LIHC cohort. **(I)** Representative IHC images of PD1, ALYREF, NR3C2 and SORBS2 staining in sorafenib sensitive- and resistant- groups from our HCC cohort. Scale bar denotes 40 μm. **(J)** Correlation analysis of sorafenib sensitivity and the expression of specified proteins. The asterisks in B, E, F and J represent the statistical p-value (*P< 0.05; **P< 0.01 and ***P< 0.001).

To ascertain the validity of the MDH score for immunotherapy evaluation, we utilized the ICGC-LIHC dataset as a test dataset to validate our above results. We first confirmed that low MDH scores exhibited significant clinical benefits and remarkably prolonged survival compared with high MDH scores in HCC (**Figure 7A**). TIDE scores were then determined for ICGC-LIHC samples, and the results suggested that high MDH scores were indeed associated with high TIDE scores and poorer immune responses in HCC (**Figure 7B**). Consistent with the TCGA analysis, HCC with high MDH scores had higher proportion of immunosuppressive infiltrating cells, including MDSCs and CAFs (**Figure 7B**). High MDH scores in ICGC-LIHC samples were also positively correlated with immune exclusion (**Figure 7B**). Moreover, high MDH scores in ICGC-LIHC samples were markedly positively correlated with high CD8 T cell and B cell levels and negatively correlated with eosinophil cell levels in HCC (**Figure S7A**), and high MDH scores caused comprehensive elevated expression of MHC molecules, costimulatory molecules, and adhesion molecules in HCC (**Figure S7B**). Consistent results also included pathway enrichment analysis, with the analysis of ICGC-LIHC samples indicating that high MDH scores were positively correlated with the cell cycle, EMT signalling and DNA replication and adversely correlated with angiogenesis (**Figure 7C**).

We further investigated whether MDH scores could predict patient response to immune checkpoint blockade therapy based on a published immunotherapy cohort (IMvigor210). As expected, a survival benefit trend was observed in the anti-PD-L1 immunotherapy cohort of patients with low MDH scores (**Figure 7D**). Moreover, patients with low MDH scores had relatively higher TMB (**Figure 7E**). Importantly, the clinical response to anti-PD-L1 immunotherapy and prominent therapeutic advantages were validated in patients with low MDH scores compared with patients with high MDH scores (**Figures 7F, G**). Moreover, patients with high MDH scores exhibited markedly higher expression of PD1, PD-L1, CD80 and CD86, which indicated that an immune-excluded phenotype may also be present in metastatic urothelial carcinoma (**Figure S7C**). Inconsistent with HCC, high MDH scores in IMvigor210 cohorts were significantly positively correlated not only with CD8 T cells and B cells but also with eosinophil cells and NK cells (**Figure S7D**). Moreover, GSVA suggested that high MDH scores were positively correlated with EMT signalling, immune checkpoints, angiogenesis and CD8 effectors and negatively correlated with the cell cycle and DNA replication (**Figure S7E**). These biological differences may be due to tumour origin and heterogeneity.

We further analysed the association between individual genes in MDH gene signatures with MDH scores and prognosis in HCC. We found that 55 genes in the MDH gene signatures were statistically significantly associated with the MDH score, 26 of which were positively correlated and 29 negatively correlated (**Figure S8A**). Moreover, 35 of the 70 genes could be used individually as prognostic genes for HCC patients, of which 12 genes were prognostic protective factors and 23 genes were adverse prognostic factors (**Figure S8B**). We further screened 8 hub genes (**Figure 7H**), three positive correlations and five negative correlations with MDH score and immune checkpoints (**Figures 7H**, **S8C**). Consistent correlation analysis results with immune checkpoints were also confirmed using ICGC-LIHC samples (**Figure S8D**). Importantly, the IHC results of our cohort validated that PD-1 expression was markedly more abundant in TKI-resistant HCC than in TKI-sensitive HCC (**Figures 7I, J**). We then validated the correlation of three Hub genes with sorafenib resistance using our HCC cohort. A positive correlation between sorafenib resistance and ALYREF protein expression, and a negative correlation between sorafenib resistance and NR3C2 or SORBS2 protein expression was obtained in our HCC cohort (**Figures 7I, J**), further supporting the relevance of the hub genes to sorafenib resistance in HCC. Furthermore, a positive correlation between PD-1 levels and ALYREF protein expression and a negative correlation between PD-1 levels and NR3C2 or SORBS2 protein expression were obtained in our HCC cohort (**Figure S8E**).

### Construction of a nomogram predicting OS in HCC based on a simplified MDH risk score

**Figure 8:**
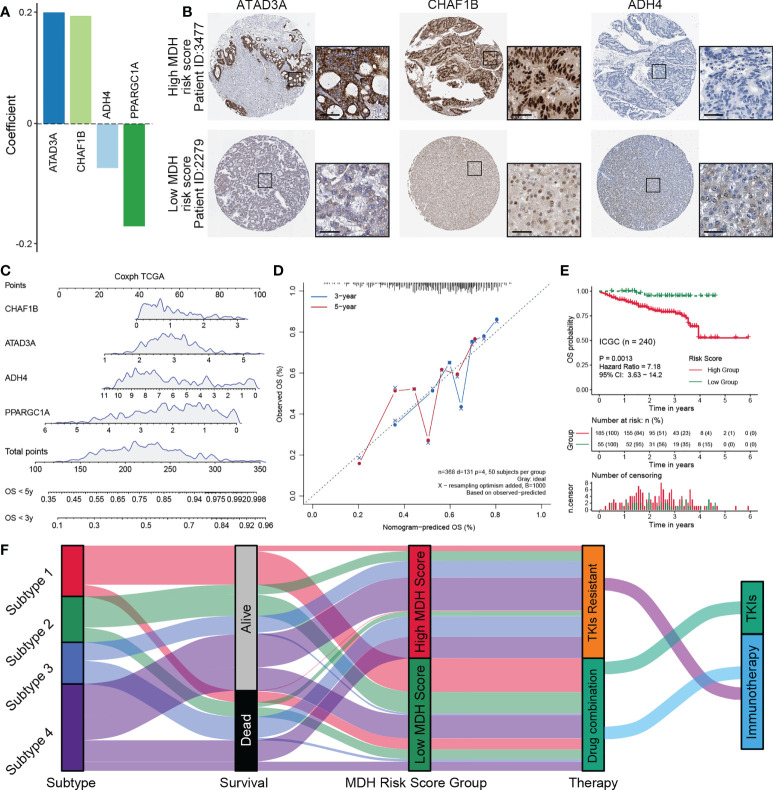
Construction of a nomogram predicting OS outcomes based on the MDH risk score in the TCGA-LIHC cohort. **(A)** Coefficients of 4 genes involved in the prognostic signatures. **(B)** Representative IHC images of ATAD3A, CHAF1B and ADH4 in MDH high- or low-score groups from the Human Protein Atlas. Scale bar denotes 50 μm. **(C)** Nomogram to predict the 3‐y and 5‐y overall survival of HCC patients. **(D)** Calibration curve for the comprehensive survival nomogram model in the TCGA-LIHC cohort. The dashed diagonal line represents the ideal situation, and the blue and red lines represent the 3‐y and 5‐y observed nomograms, respectively. **(E)** Survival analysis for the MDH risk score in the ICGC cohort. **(F)** Alluvial diagram showing the association of the MDH subtype with patient survival status, MDH score and individualized therapy.

To further simplify the MDH score and facilitate the clinical application of MDH scores, we applied an iterative LASSO Cox regression model (28). A 4-gene MDH risk score (**Figures 8A**, **S9A**) then was obtained, MDH Risk score=0.19961×ATAD3A+0.19332×CHAF1B-0.07906×ADH4-0.18327×PPARGC1A. Using TCGA-LIHC data, we confirmed that the MDH risk score can be used as an alternative diagnostic predictor of the MDH score, with an AUC of 0.659 (**Figure S9B**), and that it could also be used as a prognostic predictor for HCC (**Figure S9C**). Moreover, using the Human Protein Atlas, we confirmed that HCC with a high MDH risk score had significantly higher protein levels of ATAD3A and CHAF1B and relatively lower protein levels of ADH4 (**Figure 8B**), but PPARGC1A was not found on the website. Importantly, the correlation between the MDH score and MDH risk score was 0.68, indicating that the MDH risk score could replace the MDH score in clinical application (**Figure S9D**). Univariate and multivariate analyses were performed on the four genes, and a nomogram capable of predicting the 3- or 5-year survival probability and mortality risk of HCC patients was finally constructed (**Figures 8C**, **S9E**). The calibration curves at 3 and 5 years indicated good consistency between the prediction by the nomogram and actual overall survival outcomes (**Figure 8D**). We further calculated the MDH risk score for each HCC patient in the ICGC-LIHC dataset and compared the survival difference between the high MDH risk group and the low MDH risk group. Importantly, the results suggest that the MDH risk score can better predict the overall prognosis of HCC patients than the MDH score (**Figures 8E**, **7A**), which further proves the clinical value of the MDH risk score in predicting the outcome of HCC patients.

## Discussion

Immune checkpoint inhibition has revolutionized the cancer therapy paradigm, but effective durable responses are still observed in only a minority of patients, sometimes with severe side effects (34). Although the TKI resistance mechanisms in HCC have been extensively explored, comprehensive analysis based on the transcriptomic data of TKI-resistant HCC remains lacking. Comprehensive analysis of RNA transcripts greatly contributes to tissue deconstruction, provides a clear understanding of transcriptome-specific variations across various HCC subtypes and treatment responses, elucidates the mechanisms of therapeutic resistance to TKIs and immunotherapies and identifies novel therapeutic strategies.

Unlike previous reports, the MDH subtypes presented in our study were segregated into clusters defined by distinct signalling pathways and unique immune cell compositions. These MDH subtypes facilitated the interpretation of the underlying mechanisms of TKI resistance in HCC and revealed the intrinsic connections of the tumour immune microenvironment described among each subtype. Previous studies have revealed that the biological processes, stroma, and immunological activities of the immune microenvironment in various cancers are remarkably similar (35). Notably, four MDH subtypes could also be classified into four immunological subtypes with markedly distinct immune cell infiltration characteristics. Moreover, the observed MDH subtypes share multiple similarities with immune clusters identified in previous studies, reflecting or expanding those patterns. The stromal components and cytokines classified subtype 1 as an immune desert. Notably, the prognosis of subtype 1 was relatively good compared to subtype 2/3/4, which may be associated with marked suppression of oncogenic signalling pathways and effective response to TKIs in subtype 1 of HCC. In addition, subtypes 2/3/4 all had abundant immune cell infiltration and cytokine expression, which were strikingly separated by stromal activation level, immunosuppressive profile and mutational burden. Subtype 4 exhibits a consistent immune-activated phenotype accompanied by massive immune cell infiltration. However, subtype 3 clearly displayed a similar pattern to the immune-excluded phenotype, with marked stromal cell infiltration and activation and the worst prognosis in HCC patients. Subtype 2 showed similarities to the immune-enriched subtype but also had an angiogenic, fibrotic phenotype and was therefore classified as at the borderline between immune-activated and immune-excluded. The overall survival of HCC patients also reflects that the prognosis of subtype 4 is inferior to that of subtype 2. Our proposed MDH typing differs from previous ideas of cancer immunograms or cancer-immune set points, providing new insights for drug selection and mechanism exploration in HCC.

Immune checkpoint therapy is currently only recommended as the first-line treatment for HCC in clinical practice (36), but individualized advice is not explicitly proposed. Precision therapy can remarkably improve patient outcomes (37); thus, *a priori* identification of responders is urgently needed. Here, we further proposed an MDH score to predict the response to immunotherapy. Our study revealed that immune cell infiltration was more abundant in HCC with high MDH scores, and a high MDH score was significantly positively correlated with high expression of PD1, PDL1, CD80 and CD86. However, HCC with high MDH scores responded poorly to immune checkpoint blockade, which was mainly associated with the infiltration of stromal cells. Therefore, immune checkpoint blockade with concomitant inhibition of stromal cell activity or stromal signalling may be a beneficial therapeutic strategy for HCC patients with high MDH scores. Additionally, transcriptome profiling and clinical validation suggested that HCC patients with low MDH scores could benefit from TKIs and immunotherapy. Notably, patients with low MDH scores mostly had subtype 1, with an immune desert phenotype, suggesting that immune cell infiltration is not the only reference factor affecting the immune response and patient prognosis. Our results suggest that the TMB of subtype 1 was significantly higher than that of other subtypes, while stromal cell infiltration was significantly lower than that of other subtypes. Meanwhile, patients with low MDH scores also presented with higher eosinophilic infiltration and microsatellite instability. These results indicate that a comprehensive assessment of various markers, rather than individual factors or cells, would more accurately reflect the complexity and dynamics of the immune microenvironment and better describe its prognosis. Our proposed MDH score for predicting the efficacy of immunotherapy has been validated with different datasets and our HCC patient samples, which also indicates that our MDH score is superior in partial predictive performance to assessing tumor immunotherapy response based only on a single index.

Although multiomics studies have become more accessible and widespread over the past decade (38, 39), clinical tools that can be applied to medication decision-making for HCC patients are still lacking. To address this demand, we constructed an MDH risk score and nomogram using an iterative LASSO Cox regression model, which greatly reduces clinical effort, simplifies the prediction process and is worthy of large-scale clinical application. More importantly, MDH risk scores retain the predictive abilities of the MDH score, providing a global summary of all potential targetable alterations and mechanisms for characterizing each HCC, further providing a simple and reasonable selection for personalized treatment. However, our study also has certain limitations. Since immunotherapy has only been applied to the systemic treatment of HCC in the past two years, we did not have enough HCC samples to verify the validity of the MDH score in predicting response to immunotherapy. Thus, we only applied a public dataset containing clinical outcomes of immunotherapy for validation and analyzed the correlation of huh genes using our HCC samples. Next, we will conduct prospective clinical trials to further explore the clinical significance of MDH score and MDH risk score.

## Conclusions

In conclusion, as shown in **Figures 1A**, **8F**, this study proposed a new subtyping model for HCC and deeply investigated the underlying mechanisms of TKI resistance and the cellular infiltration characteristics of the tumour microenvironment. The MDH score might help to evaluate individualized therapy and overall survival outcomes for HCC patients, providing important clinical guidance for medication decision-making for HCC patients.

## Data availability statement

The data presented in the study are deposited in the GEO repository, accession number GSE213615.

## Ethics statement

This clinical sample study was approved by the Ethics Committee on Biomedical Research, West China Hospital of Sichuan University. The patients/participants provided their written informed consent to participate in this study. Animals received humane care, and the Institutional Animal Care and Use Committee (IACUC) approved all animal experiments.

## Author contributions

JYY, YS and LY designed the project, analyzed results and interpreted data. ZL and JSY performed the bioinformatics analysis and wrote the manuscript. ZW provided experimental assistance. JYY, YS and LY supervised the project. All authors read and approved the final manuscript.

## Funding

This work was supported by grants from the National Natural Science Foundation of China (no. 82070674), the Sichuan Province Science and Technology Department Project (no. 2019YFG0036), the 1.3.5 Project for Disciplines of Excellence, West China Hospital, Sichuan University (no. ZY2017308).

## Conflict of interest

The authors declare that the research was conducted in the absence of any commercial or financial relationships that could be construed as a potential conflict of interest.

## Publisher’s note

All claims expressed in this article are solely those of the authors and do not necessarily represent those of their affiliated organizations, or those of the publisher, the editors and the reviewers. Any product that may be evaluated in this article, or claim that may be made by its manufacturer, is not guaranteed or endorsed by the publisher.

## References

[1] Sung H Ferlay J Siegel RL Laversanne M Soerjomataram I Jemal A. Global cancer statistics 2020: GLOBOCAN estimates of incidence and mortality worldwide for 36 cancers in 185 countries. CA. Cancer J Clin (2021) 71(3):209–49. doi: 10.3322/caac.21660 33538338

[2] Akce M El-Rayes BF Bekaii-Saab TS. Frontline therapy for advanced hepatocellular carcinoma: an update. Therap. Adv Gastroenterol (2022) 15:1–12. doi: 10.1177/17562848221086126 PMC9006370 35432597

[3] Reig M Forner A Rimola J Ferrer-Fàbrega J Burrel M Garcia-Criado Á. BCLC strategy for prognosis prediction and treatment recommendation: The 2022 update. J Hepatol (2022) 76(3):681–93. doi: 10.1016/j.jhep.2021.11.018 PMC8866082 34801630

[4] Zhang CH Li M Lin YP Gao Q. Systemic therapy for hepatocellular carcinoma: Advances and hopes. Curr Gene Ther (2020) 20(2):84–99. doi: 10.2174/1566523220666200628014530 32600231

[5] Llovet JM Ricci S Mazzaferro V Hilgard P Gane E Blanc JF. Sorafenib in advanced hepatocellular carcinoma. N Engl J Med (2008) 359(4):378–90. doi: 10.1056/NEJMoa0708857 18650514

[6] Awosika J Sohal D. A narrative review of systemic treatment options for hepatocellular carcinoma: state of the art review. J Gastrointest Oncol (2022) 13(1):426–37. doi: 10.21037/jgo-21-274 PMC8899752 35284102

[7] Cheng AL Hsu C Chan SL Choo SP Kudo M. Challenges of combination therapy with immune checkpoint inhibitors for hepatocellular carcinoma. J Hepatol (2020) 72(2):307–19. doi: 10.1016/j.jhep.2019.09.025 31954494

[8] Schneider BP Jiang G Ballinger TJ Shen F Chitambar C Nanda R. BRE12-158: A postneoadjuvant, randomized phase II trial of personalized therapy versus treatment of physician's choice for patients with residual triple-negative breast cancer. J Clin Oncol (2022) 40(4):345–55. doi: 10.1200/JCO.21.01657 34910554

[9] Chalmers ZR Connelly CF Fabrizio D Gay L Ali SM Ennis R. Analysis of 100,000 human cancer genomes reveals the landscape of tumor mutational burden. Genome Med (2017) 9(1):34. doi: 10.1186/s13073-017-0424-2 28420421 PMC5395719

[10] Malta TM Sokolov A Gentles AJ Burzykowski T Poisson L Weinstein JN. Machine learning identifies stemness features associated with oncogenic dedifferentiation. Cell (2018) 173(2):338–54. doi: 10.1016/j.cell.2018.03.034 PMC5902191 29625051

[11] Liu Z Zhao Q Zuo ZX Yuan SQ Yu K Zhang Q. Systematic analysis of the aberrances and functional implications of ferroptosis in cancer. iScience (2020) 23(7):101302. doi: 10.1016/j.isci.2020.101302 32629423 PMC7334617

[12] Bindea G Mlecnik B Tosolini M Kirilovsky A Waldner M Obenauf AC. Spatiotemporal dynamics of intratumoral immune cells reveal the immune landscape in human cancer. Immunity (2013) 39(4):782–95. doi: 10.1016/j.immuni.2013.10.003 24138885

[13] Yoshihara K Shahmoradgoli M Martínez E Vegesna R Kim H Torres-Garcia W. Inferring tumour purity and stromal and immune cell admixture from expression data. Nat Commun (2013) 4:2612. doi: 10.1038/ncomms3612 24113773 PMC3826632

[14] Pipis M Rossor AM Laura M Reilly MM. Next-generation sequencing in charcot-Marie-Tooth disease: opportunities and challenges. Nat Rev Neurol (2019) 15(11):644–56. doi: 10.1038/s41582-019-0254-5 31582811

[15] Chen L Heikkinen L Wang C Yang Y Sun H Wong G. Trends in the development of miRNA bioinformatics tools. Brief Bioinform (2019) 20(5):1836–52. doi: 10.1093/bib/bby054 PMC7414524 29982332

[16] Bukowski K Kciuk M Kontek R. Mechanisms of multidrug resistance in cancer chemotherapy. Int J Mol Sci (2020) 21(9):3233. doi: 10.3390/ijms21093233 PMC7247559 32370233

[17] Llovet JM Lencioni R. mRECIST for HCC: Performance and novel refinements. J Hepatol (2020) 72(2):288–306. doi: 10.1016/j.jhep.2019.09.026 31954493 PMC12452114

[18] Yuan J Yin Z Tan L Zhu W Tao K Wang G. Interferon regulatory factor-1 reverses chemoresistance by downregulating the expression of p-glycoprotein in gastric cancer. Cancer Lett (2019) 457:28–39. doi: 10.1016/j.canlet.2019.05.006 31078735

[19] Okada S Vaeteewoottacharn K Kariya R. Application of highly immunocompromised mice for the establishment of patient-derived xenograft (PDX) models. Cells (2019) 8(8):889. doi: 10.3390/cells8080889 PMC6721637 31412684

[20] Tan L Yuan J Zhu W Tao K Wang G Gao J. Interferon regulatory factor-1 suppresses DNA damage response and reverses chemotherapy resistance by downregulating the expression of RAD51 in gastric cancer. Am J Cancer Res (2020) 10(4):1255–70. PMC7191096 32368400

[21] Sherman BT Hao M Qiu J Jiao X Baseler MW Lane HC. DAVID: a web server for functional enrichment analysis and functional annotation of gene lists (2021 update). Nucleic Acids Res (2022) 50(W1):W216–21. doi: 10.1093/nar/gkac194 PMC9252805 35325185

[22] Subramanian A Tamayo P Mootha VK Mukherjee S Ebert BL Gillette MA. Gene set enrichment analysis: a knowledge-based approach for interpreting genome-wide expression profiles. Proc Natl Acad Sci USA (2005) 102(43):15545–50. doi: 10.1073/pnas.0506580102 PMC1239896 16199517

[23] Hänzelmann S Castelo R Guinney J. GSVA: gene set variation analysis for microarray and RNA-seq data. BMC Bioinf (2013) 14:7. doi: 10.1186/1471-2105-14-7 PMC3618321 23323831

[24] Mariathasan S Turley SJ Nickles D Castiglioni A Yuen K Wang Y. TGFβ attenuates tumour response to PD-L1 blockade by contributing to exclusion of T cells. Nature (2018) 554(7693):544–8. doi: 10.1038/nature25501 PMC6028240 29443960

[25] Charoentong P Finotello F Angelova M Mayer C Efremova M Rieder D. Pan-cancer immunogenomic analyses reveal genotype-immunophenotype relationships and predictors of response to checkpoint blockade. Cell Rep (2017) 18(1):248–62. doi: 10.1016/j.celrep.2016.12.019 28052254

[26] Ben Salem K Ben Abdelaziz A. Principal component analysis (PCA). Tunis Med (2021) 99(4):383–9. PMC8734479 35244921

[27] Jiang P Gu S Pan D Fu J Sahu A Hu X. Signatures of T cell dysfunction and exclusion predict cancer immunotherapy response. Nat Med (2018) 24(10):1550–8. doi: 10.1038/s41591-018-0136-1 PMC6487502 30127393

[28] Zhao Z Liu H Zhou X Fang D Ou X Ye J. Necroptosis-related lncRNAs: Predicting prognosis and the distinction between the cold and hot tumors in gastric cancer. J Oncol (2021) 2021:6718443. doi: 10.1155/2021/6718443 34790235 PMC8592775

[29] Sangro B Sarobe P Hervás-Stubbs S Melero I. Advances in immunotherapy for hepatocellular carcinoma. Nat Rev Gastroenterol Hepatol (2021) 18(8):525–43. doi: 10.1038/s41575-021-00438-0 PMC8042636 33850328

[30] Samstein RM Lee CH Shoushtari AN Hellmann MD Shen R Janjigian YY. Tumor mutational load predicts survival after immunotherapy across multiple cancer types. Nat Genet (2019) 51(2):202–6. doi: 10.1038/s41588-018-0312-8 PMC6365097 30643254

[31] Chen DS Mellman I. Elements of cancer immunity and the cancer-immune set point. Nature (2017) 541:321–30. doi: 10.1038/nature21349 28102259

[32] Gajewski TF Woo SR Zha Y Spaapen R Zheng Y Corrales L. Cancer immunotherapy strategies based on overcoming barriers within the tumor microenvironment. Curr Opin Immunol (2013) 25:268–76. doi: 10.1016/j.coi.2013.02.009 23579075

[33] Yao HY Tseng KW Nguyen HT Kuo CT Wang HC. Hyperspectral ophthalmoscope images for the diagnosis of diabetic retinopathy stage. J Clin Med (2020) 9(6):1613. doi: 10.3390/jcm9061613 PMC7356238 32466524

[34] Khan M Arooj S Wang H. NK cell-based immune checkpoint inhibition. Front Immunol (2020) 11:167. doi: 10.3389/fimmu.2020.00167 32117298 PMC7031489

[35] Lei X Lei Y Li JK Du WX Li RG Yang J. Immune cells within the tumor microenvironment: Biological functions and roles in cancer immunotherapy. Cancer Lett (2020) 470:126–33. doi: 10.1016/j.canlet.2019.11.009 31730903

[36] Xu F Jin T Zhu Y Dai C. Immune checkpoint therapy in liver cancer. J Exp Clin Cancer Res (2018) 37(1):110. doi: 10.1186/s13046-018-0777-4 29843754 PMC5975687

[37] Dutta AK Alberge JB Sklavenitis-Pistofidis R Lightbody ED Getz G Ghobrial IM. Single-cell profiling of tumour evolution in multiple myeloma - opportunities for precision medicine. Nat Rev Clin Oncol (2022) 19(4):223–36. doi: 10.1038/s41571-021-00593-y 35017721

[38] Bagaev A Kotlov N Nomie K Svekolkin V Gafurov A Isaeva O. Conserved pan-cancer microenvironment subtypes predict response to immunotherapy. Cancer Cell (2021) 39(6):845–65. doi: 10.1016/j.ccell.2021.04.014 34019806

[39] Liu Z Liu Y Qian L Jiang S Gai X Ye S. A proteomic and phosphoproteomic landscape of KRAS mutant cancers identifies combination therapies. Mol Cell (2021) 81(19):4076–90. doi: 10.1016/j.molcel.2021.07.021 34375582

