# Supplementary material for: A novel medication decision gene signature predicts response to individualized therapy and prognosis outcomes in hepatocellular carcinoma patients
Source: Front Immunol. 2022 Oct 7;13:990571. doi: 10.3389/fimmu.2022.990571 (PMC9585274; doi:10.3389/fimmu.2022.990571)
Supplement: Supplementary file 1 [file DataSheet_1.docx]

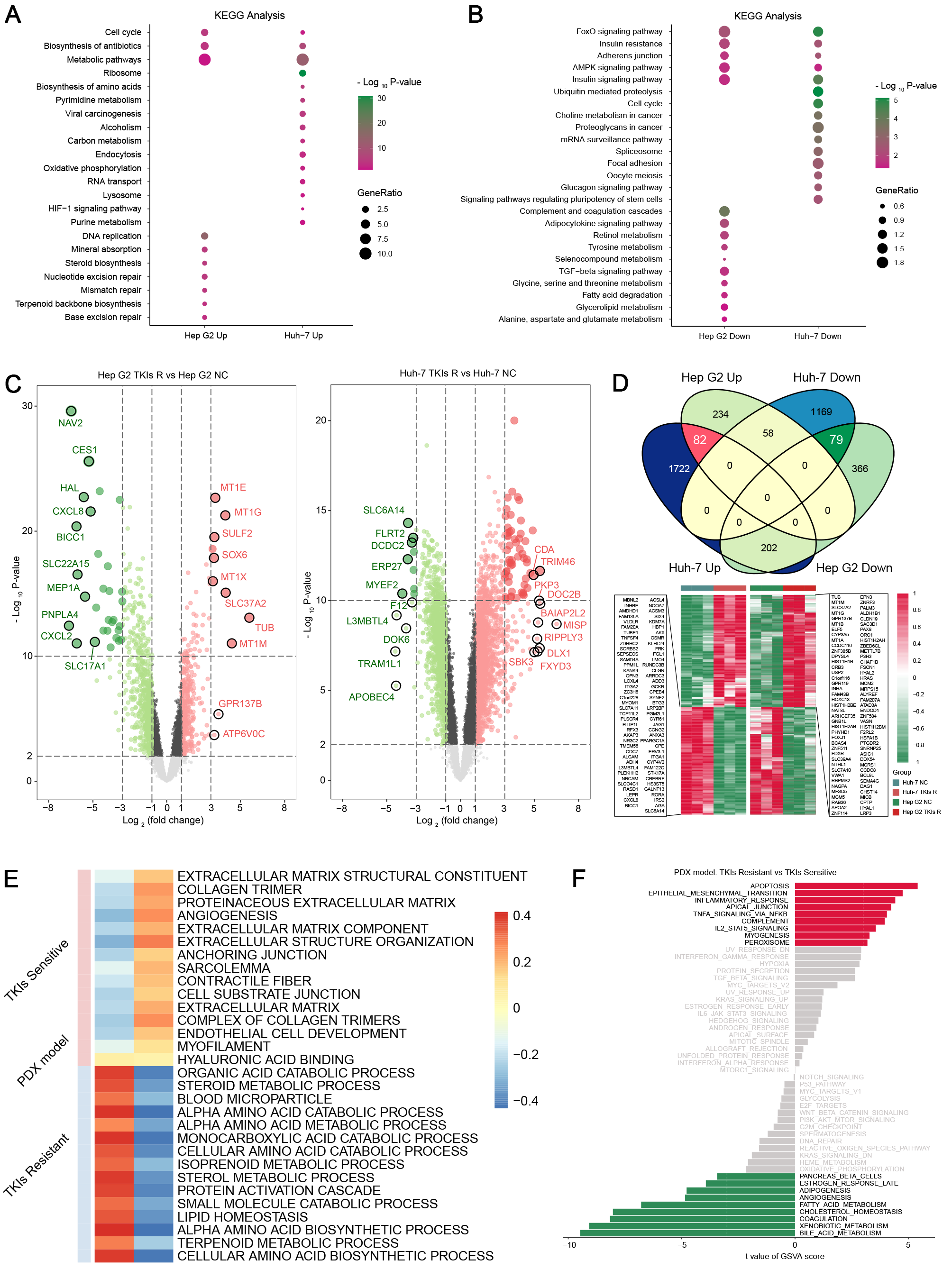


**Supplementary Figure 1.** The pathway enrichment analysis of sorafenib-resistant HCC. (A-B) KEGG enrichment analysis of up- and downregulated genes in sorafenib-resistant HCC cell lines. The asterisks in B and C represent the statistical p-value (**P < 0.01). (C) The differentially expressed genes (DEGs) in parental HCC cells and corresponding HCC sorafenib-resistant cells are visualized in volcano plots. The red and green colours indicate high and low expression, respectively. (D) Venn diagram of MDH gene signatures acquired from sorafenib-resistant HCC cell lines. (E) GSEA enrichment analysis of DEGs from the sorafenib-resistant PDX model. (F) GSVA enrichment analysis of DEGs from the sorafenib-resistant PDX model.


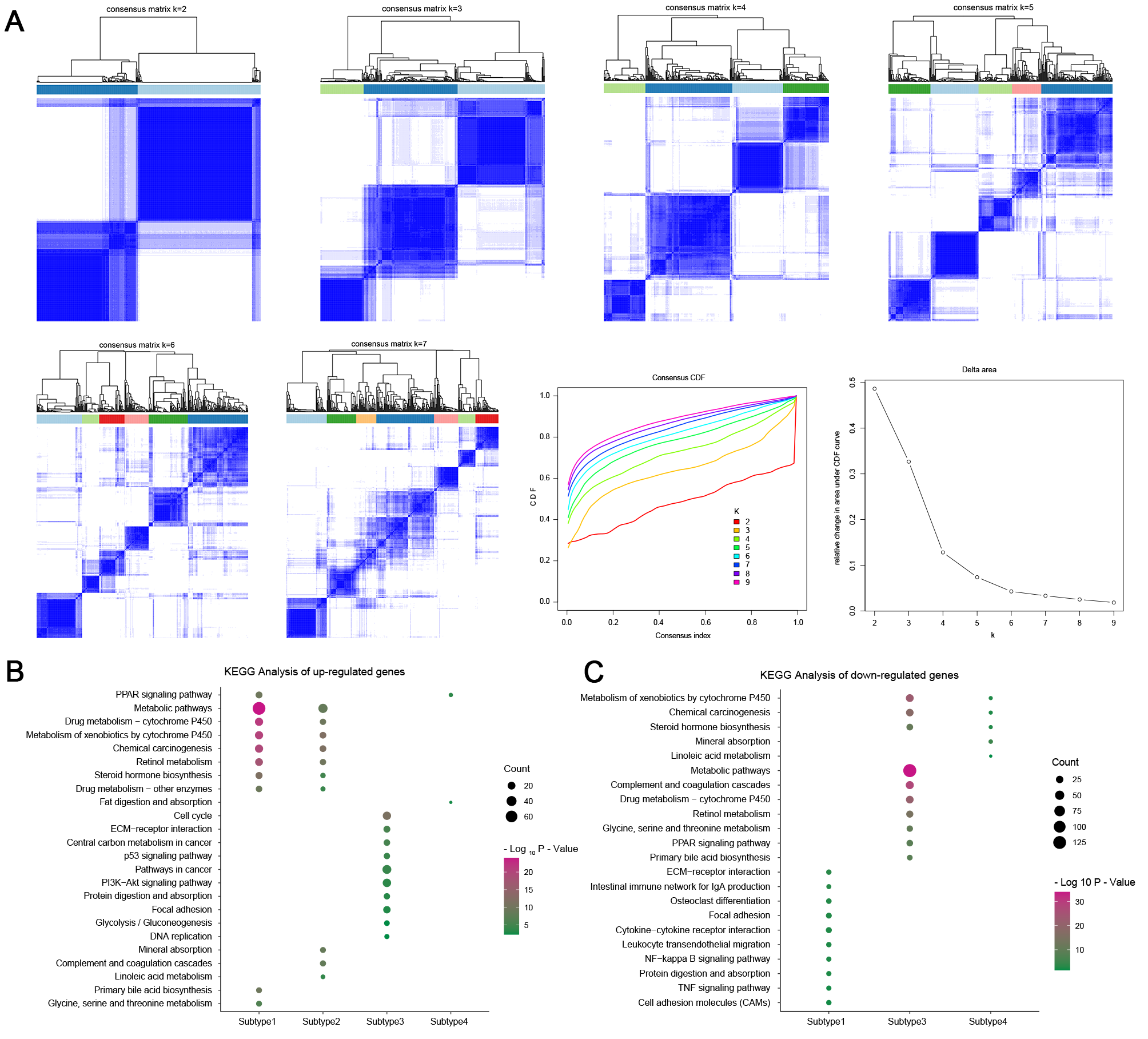


**Supplementary Figure 2.** (A) Consensus matrices of the TCGA-LIHC cohort for k = 2-6. (B-C) KEGG enrichment analysis of up-and downregulated genes in distinct MDH subtypes of the TCGA-LIHC cohort.


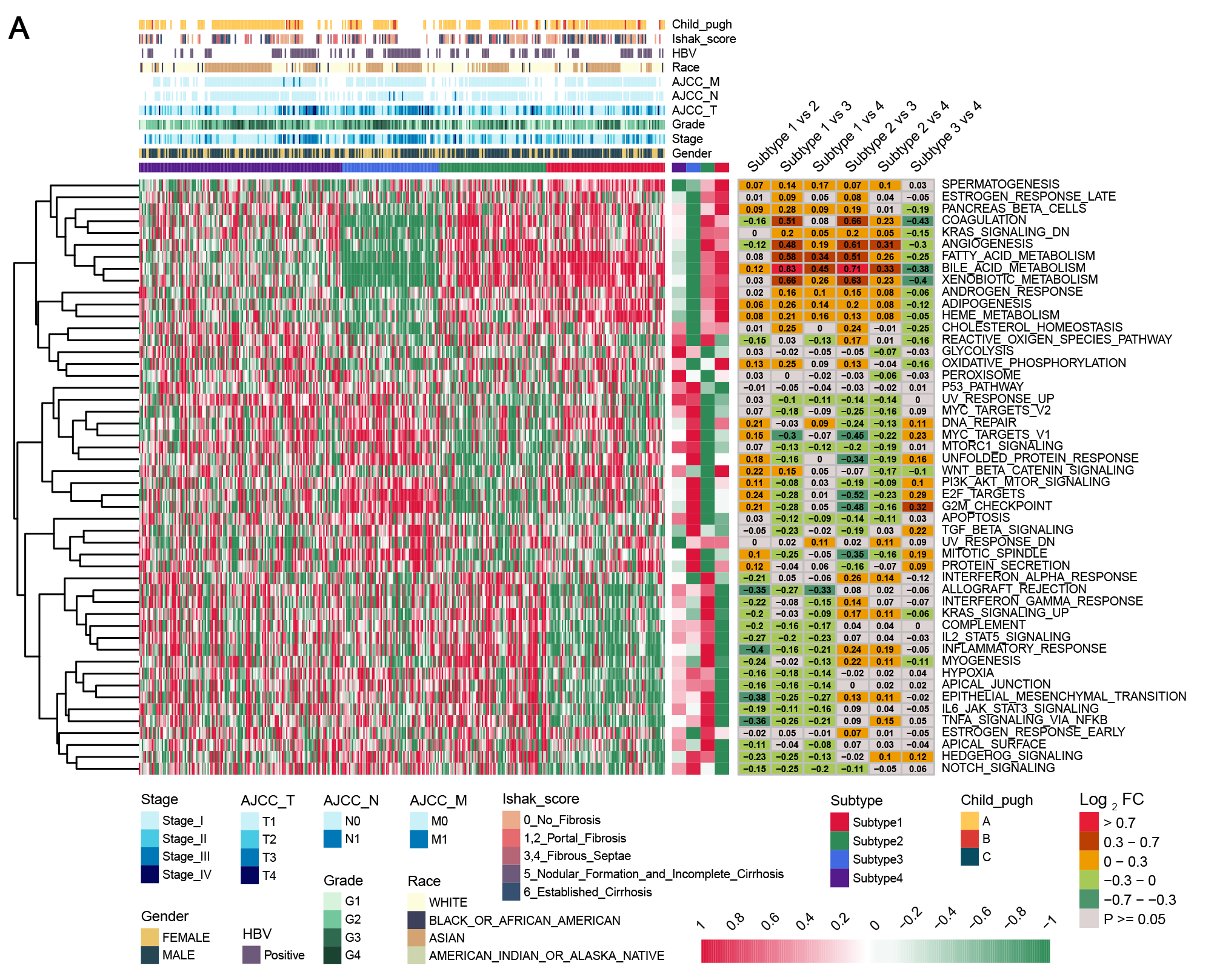


**Supplementary Figure 3**. Analysis of the transcriptional profiles of distinct MDH subtypes in the TCGA-LIHC cohort. (A) Unsupervised clustering of distinct MDH subtypes in the TCGA-LIHC cohort.


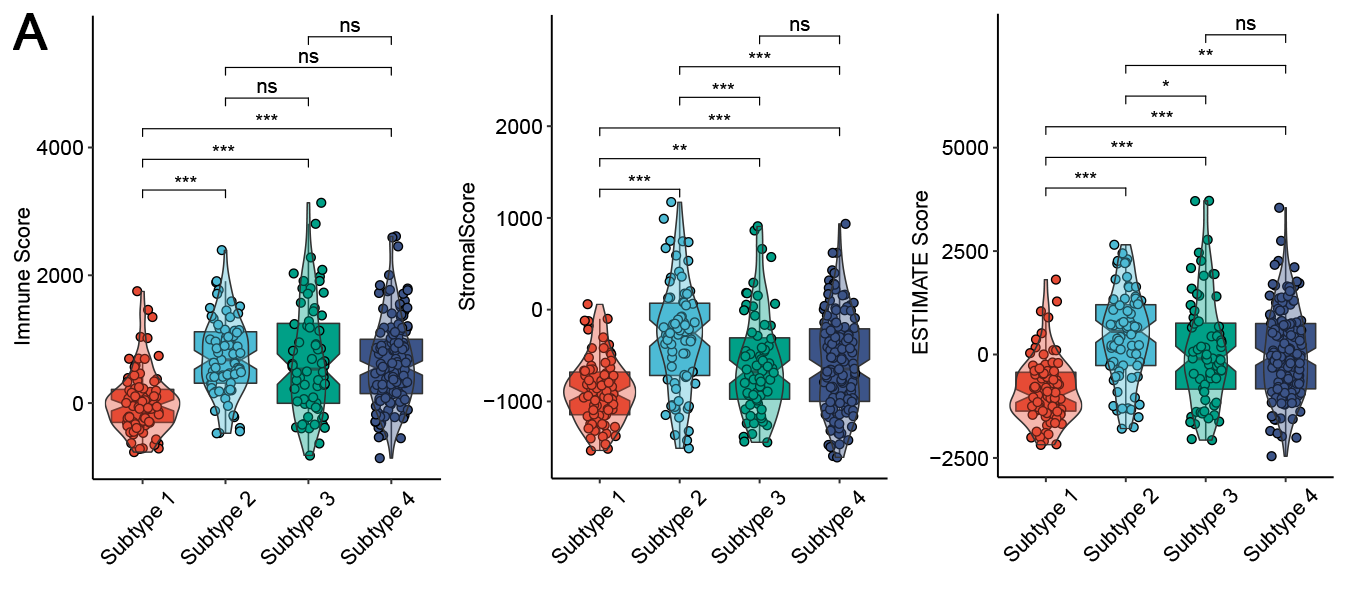


**Supplementary Figure 4**. The immune infiltration analysis of distinct MDH subtypes. (A) Immune cell infiltration score, stromal cell infiltration score and ESTIMATE score in distinct MDH subtypes of the TCGA-LIHC cohort based on the ESTIMATE algorithm. The asterisks in B-C represent the statistical p-value (*P < 0.05; **P < 0.01; ***P < 0.001, ns, no significance).


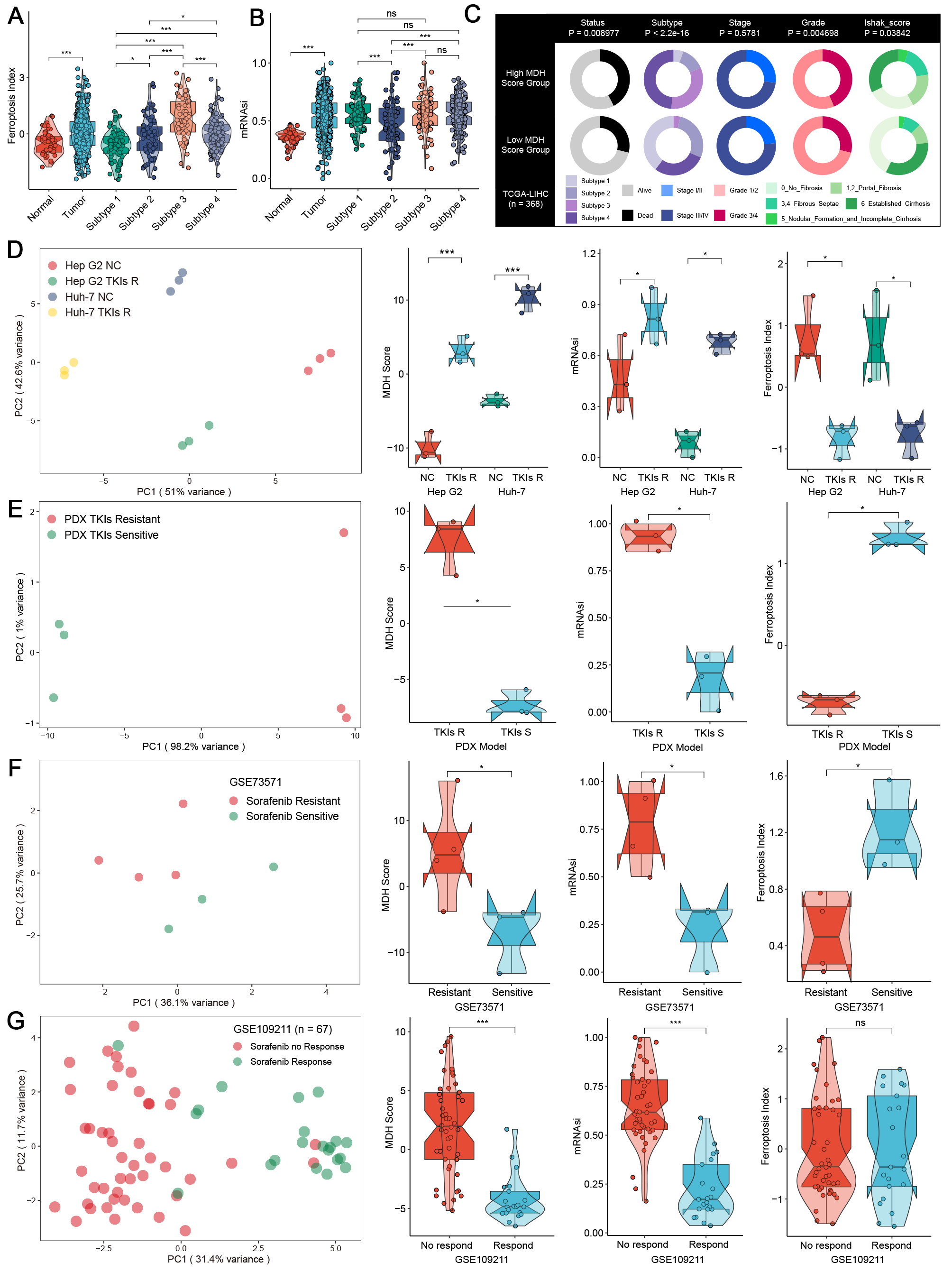


**Supplementary Figure 5**. The MDH score predicts TKI response in HCC. (A) Ferroptosis index in distinct MDH subtypes of the TCGA-LIHC cohort. (B) mRNAsi score in distinct MDH subtypes of the TCGA-LIHC cohort. (C) Clinical characterization in the MDH high- and low-score groups, and the chi-square test was used to calculate significant differences. (D) Principal component analysis for the transcriptome profiles in parental HCC cells and corresponding sorafenib-resistant HCC cells. MDH score, mRNAsi score and ferroptosis index in parental HCC cells and corresponding sorafenib-resistant HCC cells. (E) Principal component analysis for the transcriptome profiles in the sorafenib-resistant PDX model. MDH score, mRNAsi score and ferroptosis index in the sorafenib-resistant PDX model. (F-G) Principal component analysis for the transcriptome profiles in GSE73571 and GSE109211. MDH score, mRNAsi score and ferroptosis index in GSE73571 and GSE109211. The asterisks in A-B and D-G represent the statistical p-value (*P < 0.05; ***P < 0.001; ****P < 0.0001; ns, no significance).


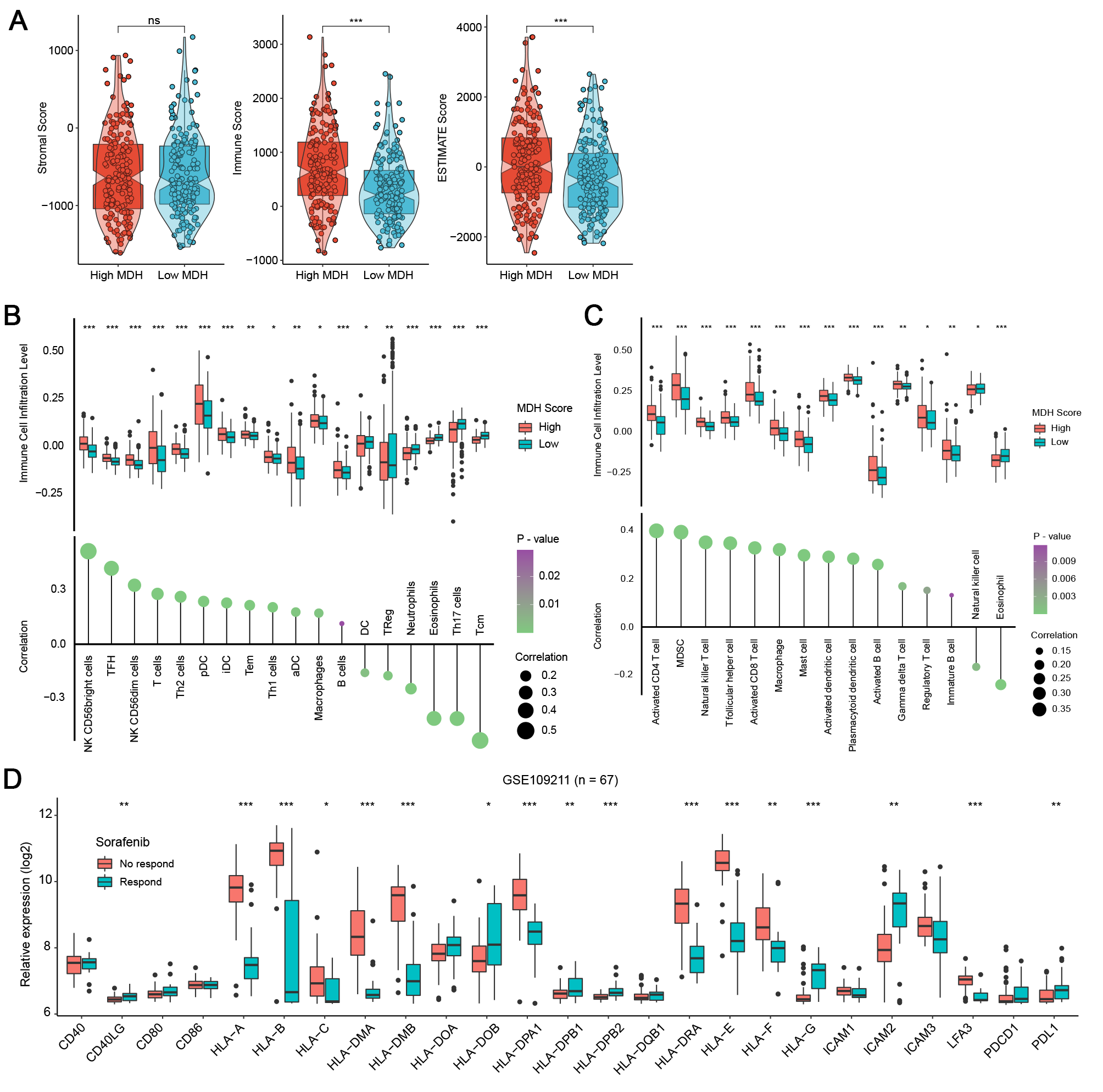


**Supplementary Figure 6**. Figure S7 Immune infiltration analysis of HCC with distinct MDH scores from the TCGA-LIHC cohort. (A) Immune cell infiltration score, stromal cell infiltration score and ESTIMATE score in the MDH high- or low-score groups of the TCGA-LIHC cohort based on the ESTIMATE algorithm. (B-C) Analysis of immune cell infiltration levels and correlation with immune cell infiltration in MDH high- or low-score groups of the TCGA-LIHC cohort based on two previously published immune cell gene signatures. The upper and lower ends of the boxes represent the interquartile range of values. The lines in the boxes represent the median value, and black dots show outliers. (D) Differences in the expression of MHC molecules, costimulatory molecules and adhesion molecules in the sorafenib response and nonresponse groups of the GSE109211 cohort. The upper and lower ends of the boxes represent the interquartile range of values. The lines in the boxes represent the median value, and black dots show outliers. The asterisks represent the statistical p-value (*P < 0.05; **P < 0.01; ***P < 0.001; ns, no significance).


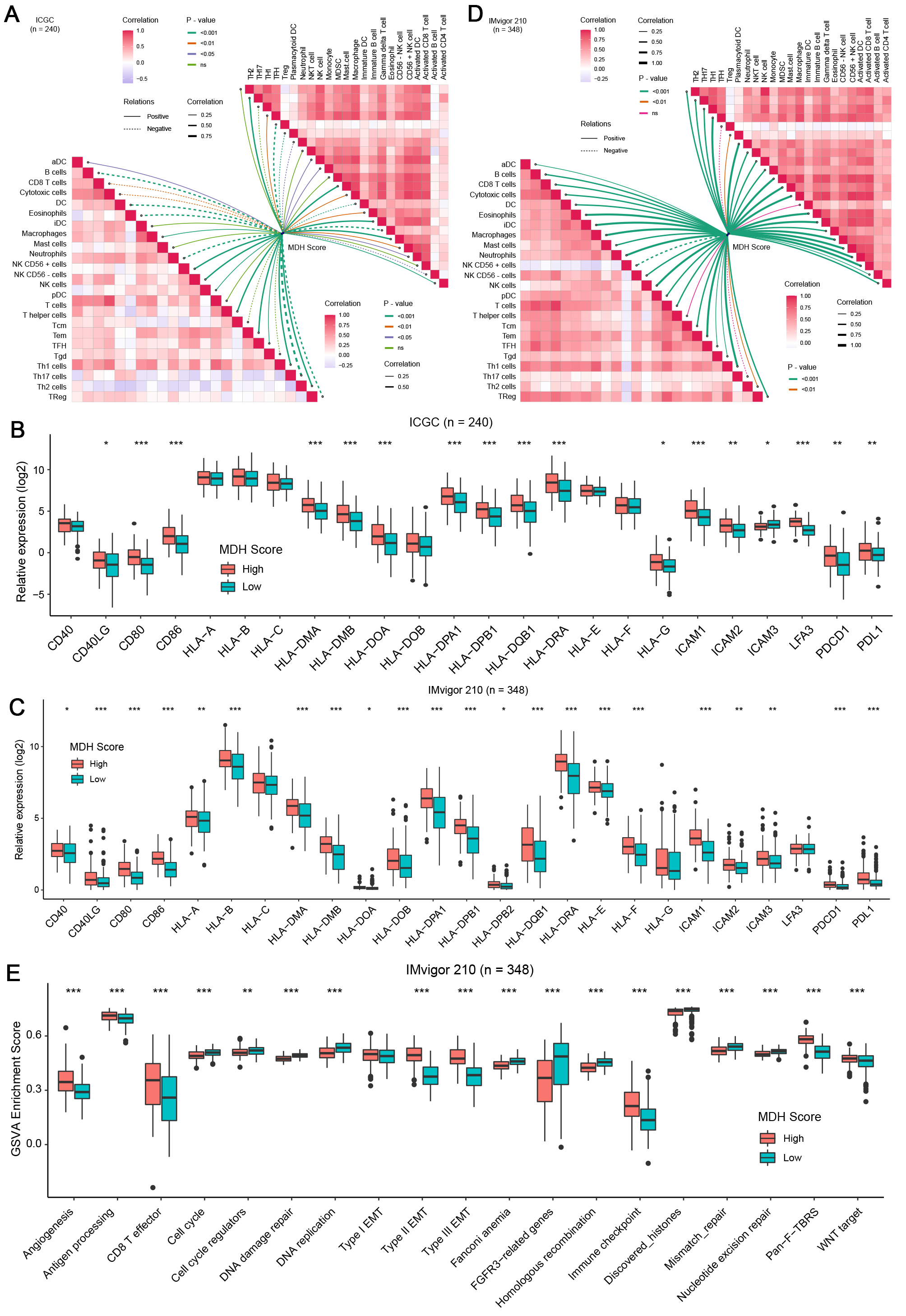


**Supplementary Figure 7**. Validation of the MDH score for the immunotherapy response based on a public cohort. (A) Correlation analysis of the MDH score with previously published immune cell gene signatures in the ICGC-LIHC cohort. (B) Differences in the expression of MHC molecules, costimulatory molecules and adhesion molecules in the MDH high- or low-score groups of the ICGC-LIHC cohort. The upper and lower ends of the boxes represent the interquartile range of values. The lines in the boxes represent the median value, and black dots show outliers. (C) Differences in the expression of MHC molecules, costimulatory molecules and adhesion molecules in the MDH high- or low-score groups of the IMvigor210 cohort. The upper and lower ends of the boxes represent the interquartile range of values. The lines in the boxes represent the median value, and black dots show outliers. (D) Correlation analysis of the MDH score with previously published immune cell gene signatures in the IMvigor210 cohort. (E) Differences in the expression of known signatures, including stromal activation-related signatures, tumour promotion-related signatures and immune activation-related signatures, in the MDH high- or low-score groups of the IMvigor210 cohort. The upper and lower ends of the boxes represent the interquartile range of values. The lines in the boxes represent the median value, and black dots show outliers. The asterisks in B-C and E represent the statistical p-value (*P < 0.05; **P < 0.01; ***P < 0.001).


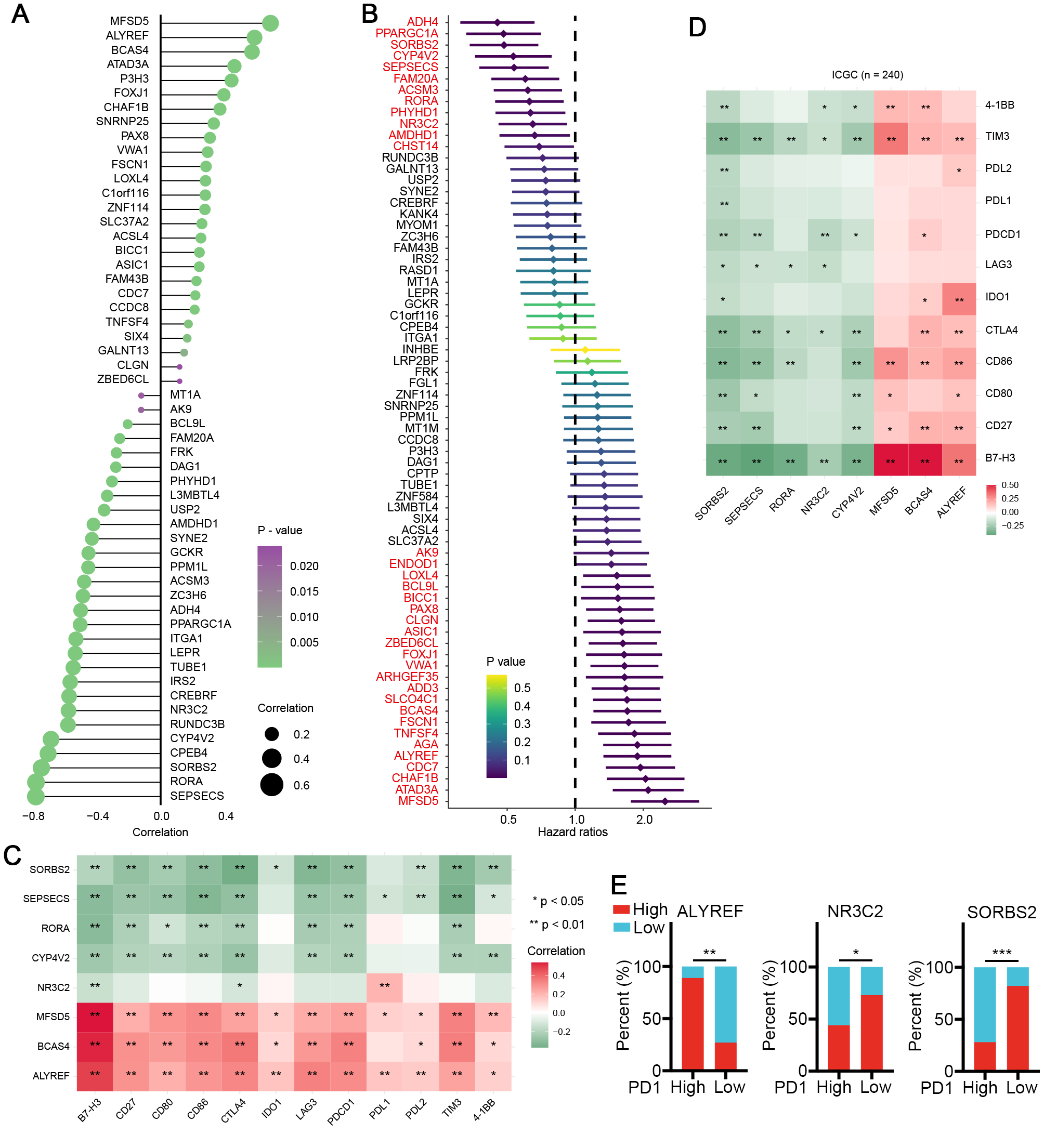


**Supplementary Figure 8**. Screening of Hub genes of the MDH gene signatures. (A) Correlation analysis of the MDH score with each gene in the MDH gene signatures of the TCGA-LIHC cohort. (B) Survival analyses for each gene in the MDH gene signatures of the TCGA-LIHC cohort. (C) Correlation analysis of 8 hub genes in MDH gene signatures with immune checkpoints in the TCGA-LIHC cohort. (D) Correlation analysis of 8 hub genes in MDH gene signatures with immune checkpoints in the ICGC-LIHC cohort. (E) Correlation analysis of the protein expression of PD1 and ALYREF, NR3C2 and SORBS2. The asterisks in C-E represent the statistical p-value (*P < 0.05; **P < 0.01; ***P < 0.001).


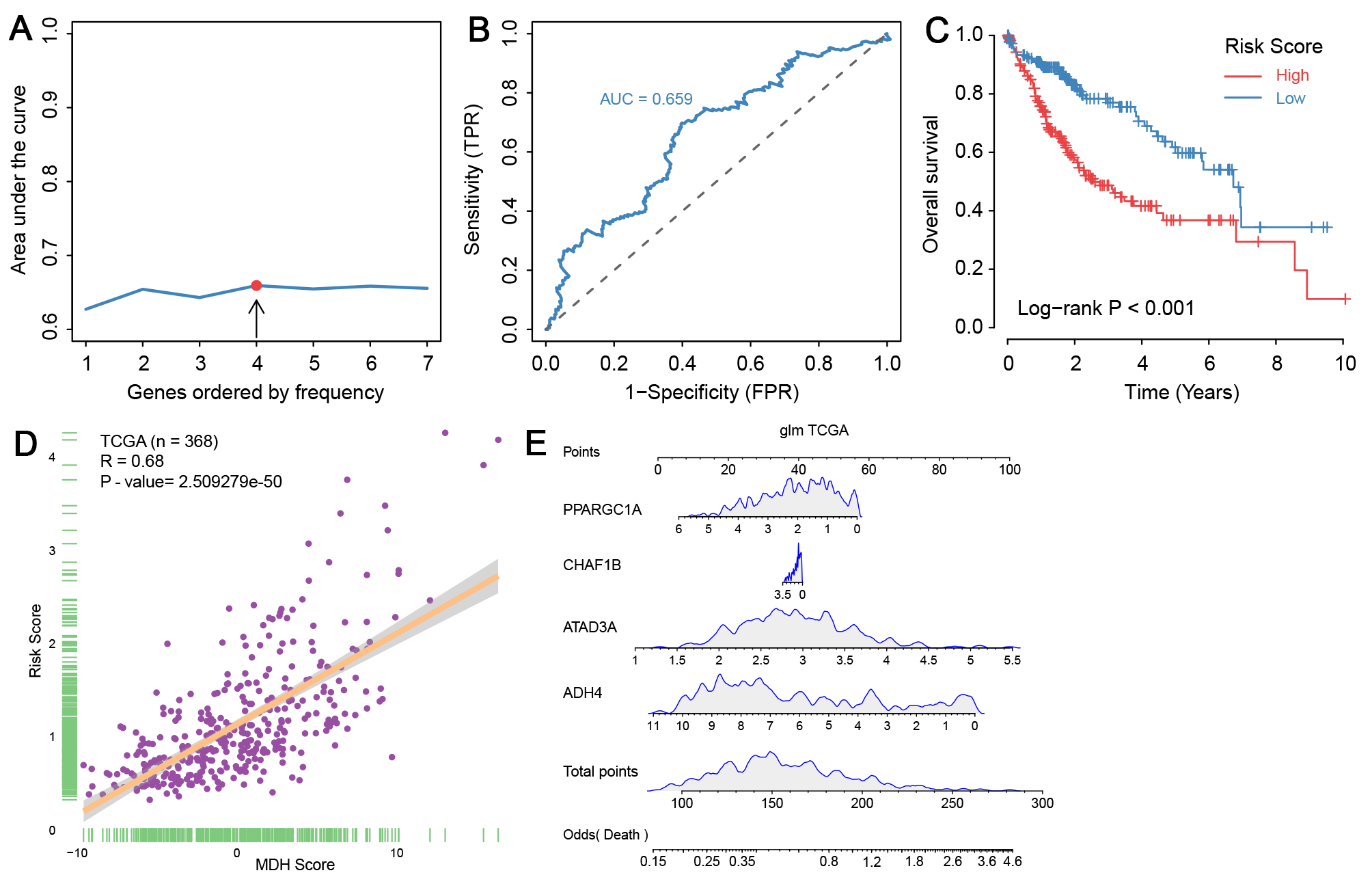


**Supplementary Figure 9**. Construction of a nomogram predicting OS outcomes based on the MDH risk score. (A-C) Iteration LASSO constructed a prognostic signature with 4 genes. The AUC for 5 years was 0.659, and the survival curve for the risk score is shown in the right panel. (D) Correlation analysis between the MDH score and risk score. (E) Nomogram to predict the mortality risk of HCC patients.

**Table S1 The clinicopathological parameters of HCC patients in this study.**

| Clinical features |  | PR (n=20) | PD (n=20) | P-value |
| --- | --- | --- | --- | --- |
| BCLC stage | A | 13 | 14 | 0.736 |
|  | B | 7 | 6 |  |
| Lymph node metastasis | No | 15 | 11 | 0.185 |
|  | Yes | 5 | 9 |  |
| Tumor differentiation | Poor | 14 | 16 | 0.465 |
|  | Well | 6 | 4 |  |
| Liver cirrhosis | No | 8 | 9 | 0.749 |
|  | Yes | 12 | 11 |  |
| Number of HCC | 1 | 16 | 18 | 0.376 |
|  | >1 | 4 | 2 |  |
| HBsAg | - | 2 | 2 | 1.000 |
|  | + | 18 | 18 |  |
| HBV DNA | - | 14 | 16 | 0.465 |
|  | + | 6 | 4 |  |
| Child-Pugh classification | A | 16 | 14 | 0.465 |
|  | B | 4 | 6 |  |
| HGB (g/L) | | 142.700±18.212 | 139.100±18.459 | 0.538 |
| TBIL (μmol/L) | | 17.015±5.747 | 18.845±10.436 | 0.496 |
| DBIL (μmol/L) | | 7.060±3.209 | 6.765±2.665 | 0.754 |
| ALT (IU/L) | | 41.700±19.129 | 36.200±16.411 | 0.335 |
| AST (IU/L) | | 89.650±114.394 | 57.700±42.579 | 0.249 |
| ALB (g/L) | | 41.460±6.829 | 40.165±6.462 | 0.542 |
| UREA (mmol/L) | | 4.817±1.476 | 5.440±1.711 | 0.225 |
| CREA (μmol/L) | | 75.660±14.442 | 72.230±21.849 | 0.562 |

PR: partial response; PD: progressive disease; HGB: hemoglobin; CREA: creatinine; ALB: albumin; TBIL: total bilirubin; DBIL: direct bilirubin; AST: aspartate aminotransferase; ALT: alanine aminotransferase.

**Table S2 Primary antibodies used in this study.**

| **Antigens** | **Manufacturer** | **Catalog Number** | **Application** |
| --- | --- | --- | --- |
| PD1 | ASGB-Bio | TA806806 | 1:200 for IHC |
| ALYREF | Proteintech | 16690-1-AP | 1:100 for IHC |
| NR3C2 | Proteintech | 21854-1-AP | 1:50 for IHC |
| SORBS2 | Proteintech | 24643-1-AP | 1:100 for IHC |

IHC, immunohistochemistry.

**Table S3 Basic information of datasets included in this study.**

| **Data base** | **Series Number** | **Disease Type** | **Total** | **Region** | **Version** |
| --- | --- | --- | --- | --- | --- |
| GEO | GSE109211 | Liver cancer | 67 | Spain | 2018 |
| GEO | GSE73571 | Liver cancer | 7 | Spain | 2018 |
| TCGA | LIHC | Liver cancer | 368 | USA | 2019 |
| ICGC | LIHC | Liver cancer | 240 | International | 2019 |
| Imvigor 210 | mUC | metastatic urothelial cancer | 348 | USA | 2018 |

mUC, metastatic urothelial cancer.

**Table S4 The gene sets used in this work for estimating the enrichment score in pathway and biological process activity.**

| **Gene Signature** | **Genes** | **Gene Signature** | **Genes** |
| --- | --- | --- | --- |
| CD8_T_effector | CD8A | Cell_cycle | CCND3 |
| CD8_T_effector | CXCL10 | Cell_cycle | CCNE1 |
| CD8_T_effector | CXCL9 | Cell_cycle | CCNE2 |
| CD8_T_effector | GZMA | Cell_cycle | CCNH |
| CD8_T_effector | GZMB | Cell_cycle | CDC14A |
| CD8_T_effector | IFNG | Cell_cycle | CDC14B |
| CD8_T_effector | PRF1 | Cell_cycle | CDC16 |
| CD8_T_effector | TBX21 | Cell_cycle | CDC20 |
| DNA_damage_repair | ALKBH2 | Cell_cycle | CDC23 |
| DNA_damage_repair | ALKBH3 | Cell_cycle | CDC25A |
| DNA_damage_repair | APEX1 | Cell_cycle | CDC25B |
| DNA_damage_repair | APEX2 | Cell_cycle | CDC25C |
| DNA_damage_repair | APLF | Cell_cycle | CDC26 |
| DNA_damage_repair | ATM | Cell_cycle | CDC27 |
| DNA_damage_repair | ATR | Cell_cycle | CDC45 |
| DNA_damage_repair | ATRIP | Cell_cycle | CDC6 |
| DNA_damage_repair | BLM | Cell_cycle | CDC7 |
| DNA_damage_repair | BRCA1 | Cell_cycle | CDK1 |
| DNA_damage_repair | BRCA2 | Cell_cycle | CDK2 |
| DNA_damage_repair | BRIP1 | Cell_cycle | CDK4 |
| DNA_damage_repair | CCNH | Cell_cycle | CDK6 |
| DNA_damage_repair | CDK7 | Cell_cycle | CDK7 |
| DNA_damage_repair | CETN2 | Cell_cycle | CDKN1A |
| DNA_damage_repair | CHAF1A | Cell_cycle | CDKN1B |
| DNA_damage_repair | CHEK1 | Cell_cycle | CDKN1C |
| DNA_damage_repair | CHEK2 | Cell_cycle | CDKN2A |
| DNA_damage_repair | CLK2 | Cell_cycle | CDKN2B |
| DNA_damage_repair | DCLRE1C | Cell_cycle | CDKN2C |
| DNA_damage_repair | DDB1 | Cell_cycle | CDKN2D |
| DNA_damage_repair | DDB2 | Cell_cycle | CHEK1 |
| DNA_damage_repair | DUT | Cell_cycle | CHEK2 |
| DNA_damage_repair | ENDOV | Cell_cycle | CREBBP |
| DNA_damage_repair | ERCC1 | Cell_cycle | CUL1 |
| DNA_damage_repair | ERCC2 | Cell_cycle | DBF4 |
| DNA_damage_repair | ERCC3 | Cell_cycle | E2F1 |
| DNA_damage_repair | ERCC4 | Cell_cycle | E2F2 |
| DNA_damage_repair | ERCC5 | Cell_cycle | E2F3 |
| DNA_damage_repair | ERCC6 | Cell_cycle | E2F4 |
| DNA_damage_repair | ERCC8 | Cell_cycle | E2F5 |
| DNA_damage_repair | FAN1 | Cell_cycle | EP300 |
| DNA_damage_repair | FANCA | Cell_cycle | ESPL1 |
| DNA_damage_repair | FANCB | Cell_cycle | FZR1 |
| DNA_damage_repair | FANCC | Cell_cycle | GADD45A |
| DNA_damage_repair | FANCD2 | Cell_cycle | GADD45B |
| DNA_damage_repair | FANCE | Cell_cycle | GADD45G |
| DNA_damage_repair | FANCF | Cell_cycle | GSK3B |
| DNA_damage_repair | FANCG | Cell_cycle | HDAC1 |
| DNA_damage_repair | FANCL | Cell_cycle | HDAC2 |
| DNA_damage_repair | FANCM | Cell_cycle | MAD1L1 |
| DNA_damage_repair | GTF2H1 | Cell_cycle | MAD2L1 |
| DNA_damage_repair | GTF2H2 | Cell_cycle | MAD2L2 |
| DNA_damage_repair | GTF2H3 | Cell_cycle | MCM2 |
| DNA_damage_repair | GTF2H4 | Cell_cycle | MCM3 |
| DNA_damage_repair | GTF2H5 | Cell_cycle | MCM4 |
| DNA_damage_repair | H2AFX | Cell_cycle | MCM5 |
| DNA_damage_repair | HLTF | Cell_cycle | MCM6 |
| DNA_damage_repair | HUS1 | Cell_cycle | MCM7 |
| DNA_damage_repair | LIG1 | Cell_cycle | MDM2 |
| DNA_damage_repair | LIG3 | Cell_cycle | MYC |
| DNA_damage_repair | LIG4 | Cell_cycle | ORC1 |
| DNA_damage_repair | MBD4 | Cell_cycle | ORC2 |
| DNA_damage_repair | MDC1 | Cell_cycle | ORC3 |
| DNA_damage_repair | MGMT | Cell_cycle | ORC4 |
| DNA_damage_repair | MLH1 | Cell_cycle | ORC5 |
| DNA_damage_repair | MLH3 | Cell_cycle | ORC6 |
| DNA_damage_repair | MMS19 | Cell_cycle | PCNA |
| DNA_damage_repair | MNAT1 | Cell_cycle | PKMYT1 |
| DNA_damage_repair | MPG | Cell_cycle | PLK1 |
| DNA_damage_repair | MSH2 | Cell_cycle | PRKDC |
| DNA_damage_repair | MSH3 | Cell_cycle | PTTG1 |
| DNA_damage_repair | MSH4 | Cell_cycle | PTTG2 |
| DNA_damage_repair | MSH5 | Cell_cycle | RAD21 |
| DNA_damage_repair | MSH6 | Cell_cycle | RB1 |
| DNA_damage_repair | MUTYH | Cell_cycle | RBL1 |
| DNA_damage_repair | NEIL1 | Cell_cycle | RBL2 |
| DNA_damage_repair | NEIL2 | Cell_cycle | RBX1 |
| DNA_damage_repair | NEIL3 | Cell_cycle | SFN |
| DNA_damage_repair | NHEJ1 | Cell_cycle | SKP1 |
| DNA_damage_repair | NTHL1 | Cell_cycle | SKP2 |
| DNA_damage_repair | NUDT1 | Cell_cycle | SMAD2 |
| DNA_damage_repair | OGG1 | Cell_cycle | SMAD3 |
| DNA_damage_repair | PALB2 | Cell_cycle | SMAD4 |
| DNA_damage_repair | PARP1 | Cell_cycle | SMC1A |
| DNA_damage_repair | PARP2 | Cell_cycle | SMC1B |
| DNA_damage_repair | PARP3 | Cell_cycle | SMC3 |
| DNA_damage_repair | PCNA | Cell_cycle | STAG1 |
| DNA_damage_repair | PER1 | Cell_cycle | STAG2 |
| DNA_damage_repair | PMS1 | Cell_cycle | TFDP1 |
| DNA_damage_repair | PMS2 | Cell_cycle | TFDP2 |
| DNA_damage_repair | PNKP | Cell_cycle | TGFB1 |
| DNA_damage_repair | POLB | Cell_cycle | TGFB2 |
| DNA_damage_repair | POLD1 | Cell_cycle | TGFB3 |
| DNA_damage_repair | POLE | Cell_cycle | TP53 |
| DNA_damage_repair | POLG | Cell_cycle | TTK |
| DNA_damage_repair | POLH | Cell_cycle | WEE1 |
| DNA_damage_repair | POLL | Cell_cycle | YWHAB |
| DNA_damage_repair | POLM | Cell_cycle | YWHAE |
| DNA_damage_repair | POLQ | Cell_cycle | YWHAG |
| DNA_damage_repair | PRKDC | Cell_cycle | YWHAH |
| DNA_damage_repair | RAD1 | Cell_cycle | YWHAQ |
| DNA_damage_repair | RAD17 | Cell_cycle | YWHAZ |
| DNA_damage_repair | RAD18 | Cell_cycle | ZBTB17 |
| DNA_damage_repair | RAD23A | DNA_replication | DNA2 |
| DNA_damage_repair | RAD23B | DNA_replication | FEN1 |
| DNA_damage_repair | RAD51C | DNA_replication | LIG1 |
| DNA_damage_repair | RAD9A | DNA_replication | MCM2 |
| DNA_damage_repair | RECQL4 | DNA_replication | MCM3 |
| DNA_damage_repair | RECQL5 | DNA_replication | MCM4 |
| DNA_damage_repair | RIF1 | DNA_replication | MCM5 |
| DNA_damage_repair | RNF168 | DNA_replication | MCM6 |
| DNA_damage_repair | RNF4 | DNA_replication | MCM7 |
| DNA_damage_repair | RNF8 | DNA_replication | PCNA |
| DNA_damage_repair | RPA1 | DNA_replication | POLA1 |
| DNA_damage_repair | RPA2 | DNA_replication | POLA2 |
| DNA_damage_repair | RPA3 | DNA_replication | POLD1 |
| DNA_damage_repair | RPA4 | DNA_replication | POLD2 |
| DNA_damage_repair | RRM2B | DNA_replication | POLD3 |
| DNA_damage_repair | SETMAR | DNA_replication | POLD4 |
| DNA_damage_repair | SHPRH | DNA_replication | POLE |
| DNA_damage_repair | SMUG1 | DNA_replication | POLE2 |
| DNA_damage_repair | TDP1 | DNA_replication | POLE3 |
| DNA_damage_repair | TDP2 | DNA_replication | POLE4 |
| DNA_damage_repair | TOPBP1 | DNA_replication | PRIM1 |
| DNA_damage_repair | TP53 | DNA_replication | PRIM2 |
| DNA_damage_repair | TREX1 | DNA_replication | RFC1 |
| DNA_damage_repair | UBE2A | DNA_replication | RFC2 |
| DNA_damage_repair | UBE2B | DNA_replication | RFC3 |
| DNA_damage_repair | UBE2N | DNA_replication | RFC4 |
| DNA_damage_repair | UBE2V2 | DNA_replication | RFC5 |
| DNA_damage_repair | UNG | DNA_replication | RNASEH1 |
| DNA_damage_repair | UVSSA | DNA_replication | RNASEH2A |
| DNA_damage_repair | WRN | DNA_replication | RNASEH2B |
| DNA_damage_repair | XAB2 | DNA_replication | RNASEH2C |
| DNA_damage_repair | XPA | DNA_replication | RPA1 |
| DNA_damage_repair | XPC | DNA_replication | RPA2 |
| DNA_damage_repair | XRCC1 | DNA_replication | RPA3 |
| DNA_damage_repair | XRCC4 | DNA_replication | RPA4 |
| DNA_damage_repair | XRCC5 | DNA_replication | SSBP1 |
| DNA_damage_repair | XRCC6 | Nucleotide_excision_repair | CCNH |
| Pan-F-TBRS | ACTA2 | Nucleotide_excision_repair | CDK7 |
| Pan-F-TBRS | ACTG2 | Nucleotide_excision_repair | CETN2 |
| Pan-F-TBRS | ADAM12 | Nucleotide_excision_repair | CUL4A |
| Pan-F-TBRS | ADAM19 | Nucleotide_excision_repair | CUL4B |
| Pan-F-TBRS | CNN1 | Nucleotide_excision_repair | DDB1 |
| Pan-F-TBRS | COL4A1 | Nucleotide_excision_repair | DDB2 |
| Pan-F-TBRS | CTGF | Nucleotide_excision_repair | ERCC1 |
| Pan-F-TBRS | CTPS1 | Nucleotide_excision_repair | ERCC2 |
| Pan-F-TBRS | FAM101B | Nucleotide_excision_repair | ERCC3 |
| Pan-F-TBRS | FSTL3 | Nucleotide_excision_repair | ERCC4 |
| Pan-F-TBRS | HSPB1 | Nucleotide_excision_repair | ERCC5 |
| Pan-F-TBRS | IGFBP3 | Nucleotide_excision_repair | ERCC6 |
| Pan-F-TBRS | PXDC1 | Nucleotide_excision_repair | ERCC8 |
| Pan-F-TBRS | SEMA7A | Nucleotide_excision_repair | GTF2H1 |
| Pan-F-TBRS | SH3PXD2A | Nucleotide_excision_repair | GTF2H2 |
| Pan-F-TBRS | TAGLN | Nucleotide_excision_repair | GTF2H3 |
| Pan-F-TBRS | TGFBI | Nucleotide_excision_repair | GTF2H4 |
| Pan-F-TBRS | TNS1 | Nucleotide_excision_repair | GTF2H5 |
| Pan-F-TBRS | TPM1 | Nucleotide_excision_repair | LIG1 |
| Antigen_processing_machinery | B2M | Nucleotide_excision_repair | MNAT1 |
| Antigen_processing_machinery | HLA-A | Nucleotide_excision_repair | PCNA |
| Antigen_processing_machinery | HLA-B | Nucleotide_excision_repair | POLD1 |
| Antigen_processing_machinery | HLA-C | Nucleotide_excision_repair | POLD2 |
| Antigen_processing_machinery | TAP1 | Nucleotide_excision_repair | POLD3 |
| Antigen_processing_machinery | TAP2 | Nucleotide_excision_repair | POLD4 |
| Immune_checkpoint | CD274 | Nucleotide_excision_repair | POLE |
| Immune_checkpoint | CTLA4 | Nucleotide_excision_repair | POLE2 |
| Immune_checkpoint | HAVCR2 | Nucleotide_excision_repair | POLE3 |
| Immune_checkpoint | LAG3 | Nucleotide_excision_repair | POLE4 |
| Immune_checkpoint | PDCD1 | Nucleotide_excision_repair | RAD23A |
| Immune_checkpoint | PDCD1LG2 | Nucleotide_excision_repair | RAD23B |
| Immune_checkpoint | TIGIT | Nucleotide_excision_repair | RBX1 |
| EMT_(1) | CLDN3 | Nucleotide_excision_repair | RFC1 |
| EMT_(1) | CLDN7 | Nucleotide_excision_repair | RFC2 |
| EMT_(1) | CLDN4 | Nucleotide_excision_repair | RFC3 |
| EMT_(1) | CDH1 | Nucleotide_excision_repair | RFC4 |
| EMT_(1) | VIM | Nucleotide_excision_repair | RFC5 |
| EMT_(1) | TWIST1 | Nucleotide_excision_repair | RPA1 |
| EMT_(1) | ZEB1 | Nucleotide_excision_repair | RPA2 |
| EMT_(1) | ZEB2 | Nucleotide_excision_repair | RPA3 |
| FGFR3-related_genees | FGFR3 | Nucleotide_excision_repair | RPA4 |
| FGFR3-related_genees | TP63 | Nucleotide_excision_repair | XPA |
| FGFR3-related_genees | WNT7B | Nucleotide_excision_repair | XPC |
| KEGG_discovered_histones | HIST1H2AG | Homologous_recombination | BLM |
| KEGG_discovered_histones | HIST1H2AI | Homologous_recombination | BRCA2 |
| KEGG_discovered_histones | HIST1H2BL | Homologous_recombination | EME1 |
| KEGG_discovered_histones | HIST2H2BF | Homologous_recombination | MRE11A |
| Angiogenesis | CDH5 | Homologous_recombination | MUS81 |
| Angiogenesis | SOX17 | Homologous_recombination | NBN |
| Angiogenesis | SOX18 | Homologous_recombination | POLD1 |
| Angiogenesis | TEK | Homologous_recombination | POLD2 |
| Fanconi_anemia | APITD1 | Homologous_recombination | POLD3 |
| Fanconi_anemia | ATR | Homologous_recombination | POLD4 |
| Fanconi_anemia | ATRIP | Homologous_recombination | RAD50 |
| Fanconi_anemia | BLM | Homologous_recombination | RAD51 |
| Fanconi_anemia | BRCA1 | Homologous_recombination | RAD51B |
| Fanconi_anemia | BRCA2 | Homologous_recombination | RAD51C |
| Fanconi_anemia | BRIP1 | Homologous_recombination | RAD51D |
| Fanconi_anemia | C17orf70 | Homologous_recombination | RAD52 |
| Fanconi_anemia | C19orf40 | Homologous_recombination | RAD54B |
| Fanconi_anemia | EME1 | Homologous_recombination | RAD54L |
| Fanconi_anemia | EME2 | Homologous_recombination | RPA1 |
| Fanconi_anemia | ERCC1 | Homologous_recombination | RPA2 |
| Fanconi_anemia | ERCC4 | Homologous_recombination | RPA3 |
| Fanconi_anemia | FAN1 | Homologous_recombination | RPA4 |
| Fanconi_anemia | FANCA | Homologous_recombination | SHFM1 |
| Fanconi_anemia | FANCB | Homologous_recombination | SSBP1 |
| Fanconi_anemia | FANCC | Homologous_recombination | TOP3A |
| Fanconi_anemia | FANCD2 | Homologous_recombination | TOP3B |
| Fanconi_anemia | FANCE | Homologous_recombination | XRCC2 |
| Fanconi_anemia | FANCF | Homologous_recombination | XRCC3 |
| Fanconi_anemia | FANCG | Mismatch_repair | EXO1 |
| Fanconi_anemia | FANCI | Mismatch_repair | LIG1 |
| Fanconi_anemia | FANCL | Mismatch_repair | MLH1 |
| Fanconi_anemia | FANCM | Mismatch_repair | MLH3 |
| Fanconi_anemia | HES1 | Mismatch_repair | MSH2 |
| Fanconi_anemia | MLH1 | Mismatch_repair | MSH3 |
| Fanconi_anemia | MUS81 | Mismatch_repair | MSH6 |
| Fanconi_anemia | PALB2 | Mismatch_repair | PCNA |
| Fanconi_anemia | PMS2 | Mismatch_repair | PMS2 |
| Fanconi_anemia | POLH | Mismatch_repair | POLD1 |
| Fanconi_anemia | POLI | Mismatch_repair | POLD2 |
| Fanconi_anemia | POLK | Mismatch_repair | POLD3 |
| Fanconi_anemia | POLN | Mismatch_repair | POLD4 |
| Fanconi_anemia | RAD51 | Mismatch_repair | RFC1 |
| Fanconi_anemia | RAD51C | Mismatch_repair | RFC2 |
| Fanconi_anemia | REV1 | Mismatch_repair | RFC3 |
| Fanconi_anemia | REV3L | Mismatch_repair | RFC4 |
| Fanconi_anemia | RMI1 | Mismatch_repair | RFC5 |
| Fanconi_anemia | RMI2 | Mismatch_repair | RPA1 |
| Fanconi_anemia | RPA1 | Mismatch_repair | RPA2 |
| Fanconi_anemia | RPA2 | Mismatch_repair | RPA3 |
| Fanconi_anemia | RPA3 | Mismatch_repair | RPA4 |
| Fanconi_anemia | RPA4 | Mismatch_repair | SSBP1 |
| Fanconi_anemia | SLX4 | EMT2 | AXL |
| Fanconi_anemia | STRA13 | EMT2 | FAP |
| Fanconi_anemia | TELO2 | EMT2 | LOXL2 |
| Fanconi_anemia | TOP3A | EMT2 | ROR2 |
| Fanconi_anemia | TOP3B | EMT2 | TAGLN |
| Fanconi_anemia | UBE2T | EMT2 | TWIST2 |
| Fanconi_anemia | USP1 | EMT2 | WNT5A |
| Fanconi_anemia | WDR48 | WNT_target | EFNB3 |
| Cell_cycle | ABL1 | WNT_target | MYC |
| Cell_cycle | ANAPC1 | WNT_target | TCF12 |
| Cell_cycle | ANAPC10 | WNT_target | VEGFA |
| Cell_cycle | ANAPC11 | EMT3 | FOXF1 |
| Cell_cycle | ANAPC13 | EMT3 | GATA6 |
| Cell_cycle | ANAPC2 | EMT3 | SOX9 |
| Cell_cycle | ANAPC4 | EMT3 | TWIST1 |
| Cell_cycle | ANAPC5 | EMT3 | ZEB1 |
| Cell_cycle | ANAPC7 | EMT3 | ZEB2 |
| Cell_cycle | ATM | Cell_cycle_regulators | ATM |
| Cell_cycle | ATR | Cell_cycle_regulators | CCND1 |
| Cell_cycle | BUB1 | Cell_cycle_regulators | CCNE1 |
| Cell_cycle | BUB1B | Cell_cycle_regulators | CDKN1A |
| Cell_cycle | BUB3 | Cell_cycle_regulators | CDKN2A |
| Cell_cycle | CCNA1 | Cell_cycle_regulators | E2F3 |
| Cell_cycle | CCNA2 | Cell_cycle_regulators | FBXW7 |
| Cell_cycle | CCNB1 | Cell_cycle_regulators | MDM2 |
| Cell_cycle | CCNB2 | Cell_cycle_regulators | RB1 |
| Cell_cycle | CCNB3 | Cell_cycle_regulators | TP53 |
| Cell_cycle | CCND1 | Cell_cycle | CCND2 |

**Table S5 The immune cell signature 1 used in this work to estimate the relative abundance of immune cell infiltration.**

| **Cell_Type** | **Gene_Symbol** | **Cell_Type** | **Gene_Symbol** | **Cell_Type** | **Gene_Symbol** |
| --- | --- | --- | --- | --- | --- |
| aDC | LAMP3 | Macrophages | SCG5 | T cells | TRDV3 |
| aDC | CCL1 | Macrophages | CYBB | T cells | TRAT1 |
| aDC | IDO1 | Macrophages | ME1 | T cells | BCL11B |
| aDC | OAS3 | Macrophages | GPC4 | T helper cells | UBE2L3 |
| aDC | EBI3 | Macrophages | GPC4 | T helper cells | ANP32B |
| B cells | GLDC | Macrophages | KAL1 | T helper cells | NAP1L4 |
| B cells | COCH | Macrophages | SULT1C2 | T helper cells | RPA1 |
| B cells | SLC15A2 | Macrophages | MARCO | T helper cells | SEC24C |
| B cells | CR2 | Macrophages | CCL7 | T helper cells | ITM2A |
| B cells | HLA-DOB | Macrophages | CHIT1 | T helper cells | SLC25A12 |
| B cells | SPIB | Macrophages | MSR1 | T helper cells | ASF1A |
| B cells | PNOC | Macrophages | CHI3L1 | T helper cells | ATF2 |
| B cells | BLK | Macrophages | CD84 | T helper cells | BATF |
| B cells | CD19 | Macrophages | SULT1C2 | T helper cells | GOLGA8A |
| B cells | KIAA0125 | Macrophages | PTGDS | T helper cells | ICOS |
| B cells | TNFRSF17 | Macrophages | MSR1 | T helper cells | CD28 |
| B cells | GNG7 | Macrophages | PTGDS | T helper cells | FRYL |
| B cells | CCR9 | Macrophages | SGMS1 | T helper cells | PPP2R5C |
| B cells | BLNK | Macrophages | BCAT1 | T helper cells | SRSF10 |
| B cells | ABCB4 | Macrophages | BCAT1 | T helper cells | LRBA |
| B cells | MEF2C | Macrophages | FN1 | T helper cells | FAM111A |
| B cells | MEF2C | Macrophages | MSR1 | T helper cells | NUP107 |
| B cells | IGHM | Macrophages | CXCL5 | T helper cells | PHF10 |
| B cells | TCL1A | Macrophages | CD163 | T helper cells | BORA |
| B cells | BCL11A | Macrophages | GM2A | T helper cells | DDX50 |
| B cells | MS4A1 | Macrophages | ATG7 | Tcm | SLC7A6 |
| B cells | SCN3A | Macrophages | PCOLCE2 | Tcm | AQP3 |
| B cells | MICAL3 | Macrophages | MS4A4A | Tcm | CLUAP1 |
| B cells | IGHD | Macrophages | CLEC5A | Tcm | FYB |
| B cells | IGHA1 | Macrophages | DNASE2B | Tcm | USP9Y |
| B cells | IGKC | Macrophages | COLEC12 | Tcm | TXK |
| B cells | DTNB | Macrophages | COL8A2 | Tcm | KLF12 |
| B cells | CD72 | Mast cells | ABCC4 | Tcm | CASP8 |
| B cells | IGLC1 | Mast cells | MPO | Tcm | CEP68 |
| B cells | MS4A1 | Mast cells | SCG2 | Tcm | ATM |
| B cells | QRSL1 | Mast cells | MAOB | Tcm | PCM1 |
| B cells | QRSL1 | Mast cells | VWA5A | Tcm | CDC14A |
| B cells | OSBPL10 | Mast cells | KIT | Tcm | NFATC3 |
| B cells | BCL11A | Mast cells | PTGS1 | Tcm | TIMM8A |
| B cells | KIAA0125 | Mast cells | CALB2 | Tcm | ATM |
| B cells | BACH2 | Mast cells | CPA3 | Tcm | HNRNPH1 |
| B cells | FCRL2 | Mast cells | CTSG | Tcm | CREBZF |
| B cells | IGHD | Mast cells | TPSAB1 | Tcm | TXLNGY |
| B cells | TCL1A | Mast cells | SLC18A2 | Tcm | CYLD |
| CD8 T cells | DNAJB1 | Mast cells | ADCYAP1 | Tcm | MAP3K1 |
| CD8 T cells | DNAJB1 | Mast cells | TAL1 | Tcm | DOCK9 |
| CD8 T cells | ZFP36L2 | Mast cells | SIGLEC6 | Tcm | N4BP2L2-IT2 |
| CD8 T cells | ZFP36L2 | Mast cells | SIGLEC6 | Tcm | PCNX |
| CD8 T cells | VAMP2 | Mast cells | NR0B1 | Tcm | REPS1 |
| CD8 T cells | PPP1R2 | Mast cells | CEACAM8 | Tcm | TRAF3IP3 |
| CD8 T cells | TBCC | Mast cells | HPGDS | Tcm | PHC3 |
| CD8 T cells | LEPROTL1 | Mast cells | ELANE | Tcm | NMT2 |
| CD8 T cells | CAMLG | Mast cells | HDC | Tcm | KMT2A |
| CD8 T cells | KLF9 | Mast cells | TPSB2 | Tcm | SPDYE2 |
| CD8 T cells | GADD45A | Mast cells | MS4A2 | Tcm | STX16 |
| CD8 T cells | CD8A | Mast cells | MS4A2 | Tcm | NEFL |
| CD8 T cells | ZNF91 | Mast cells | TPSAB1 | Tem | TBCD |
| CD8 T cells | PF4 | Mast cells | GATA2 | Tem | TBC1D5 |
| CD8 T cells | THUMPD1 | Mast cells | TPSAB1 | Tem | NFATC4 |
| CD8 T cells | TSC22D3 | Mast cells | VWA5A | Tem | PRKY |
| CD8 T cells | SLC16A7 | Mast cells | SIGLEC6 | Tem | CCR2 |
| CD8 T cells | GZMM | Mast cells | HPGD | Tem | LTK |
| CD8 T cells | SIK1 | Mast cells | PRG2 | Tem | CCR2 |
| CD8 T cells | RBM3 | Mast cells | PPM1H | Tem | MEFV |
| CD8 T cells | APBA2 | Mast cells | CMA1 | Tem | DDX17 |
| CD8 T cells | HAUS3 | Mast cells | LINC01140 | Tem | SND1-IT1 |
| CD8 T cells | SF1 | Mast cells | TPSAB1 | Tem | FLI1 |
| CD8 T cells | FLT3LG | Mast cells | MLPH | Tem | FLI1 |
| CD8 T cells | TMEM259 | Mast cells | SLC24A3 | Tem | AKT3 |
| CD8 T cells | ZNF609 | Mast cells | SLC24A3 | Tem | EZR |
| CD8 T cells | SRSF7 | Neutrophils | CD93 | Tem | TCRA |
| CD8 T cells | PRF1 | Neutrophils | CD93 | Tem | GDPD5 |
| CD8 T cells | TMC6 | Neutrophils | MME | TFH | STK39 |
| CD8 T cells | KAT6A | Neutrophils | MME | TFH | PTPN13 |
| CD8 T cells | AES | Neutrophils | CSF3R | TFH | CHGB |
| CD8 T cells | ZNF22 | Neutrophils | FCGR3B | TFH | TOX |
| CD8 T cells | ABT1 | Neutrophils | TECPR2 | TFH | CDK5R1 |
| CD8 T cells | CDKN2AIP | Neutrophils | FPR1 | TFH | CXCL13 |
| CD8 T cells | PRR5 | Neutrophils | BST1 | TFH | PVALB |
| CD8 T cells | LIME1 | Neutrophils | S100A12 | TFH | CXCR5 |
| CD8 T cells | PRR5 | Neutrophils | SLC22A4 | TFH | MAF |
| CD8 T cells | C12orf47 | Neutrophils | TNFRSF10C | TFH | PDCD1 |
| Cytotoxic cells | RUNX3 | Neutrophils | CYP4F3 | TFH | ICA1 |
| Cytotoxic cells | DUSP2 | Neutrophils | MGAM | TFH | LDLRAD4 |
| Cytotoxic cells | GZMA | Neutrophils | CXCR2 | TFH | MYO7A |
| Cytotoxic cells | GNLY | Neutrophils | CXCR1 | TFH | MAF |
| Cytotoxic cells | ZBTB16 | Neutrophils | FCAR | TFH | LDLRAD4 |
| Cytotoxic cells | KLRD1 | Neutrophils | CEACAM3 | TFH | TSHR |
| Cytotoxic cells | KLRD1 | Neutrophils | LILRB2 | TFH | ST8SIA1 |
| Cytotoxic cells | APBA2 | Neutrophils | FPR2 | TFH | MYO6 |
| Cytotoxic cells | GZMH | Neutrophils | FPR2 | TFH | ICA1 |
| Cytotoxic cells | RORA | Neutrophils | CEACAM3 | TFH | SMAD1 |
| Cytotoxic cells | KLRD1 | Neutrophils | TNFRSF10C | TFH | CHI3L2 |
| Cytotoxic cells | NKG7 | Neutrophils | FCAR | TFH | PASK |
| Cytotoxic cells | CTSW | Neutrophils | KCNJ15 | TFH | HIST1H4J |
| Cytotoxic cells | KLRB1 | Neutrophils | FCAR | TFH | CXCR5 |
| Cytotoxic cells | SIGIRR | Neutrophils | G0S2 | TFH | PASK |
| Cytotoxic cells | KLRF1 | Neutrophils | ALPL | TFH | MKL2 |
| Cytotoxic cells | APOL3 | Neutrophils | SLC25A37 | TFH | POMT1 |
| Cytotoxic cells | GNLY | Neutrophils | CPPED1 | TFH | MAGEH1 |
| Cytotoxic cells | SIGIRR | Neutrophils | DYSF | TFH | HEY1 |
| DC | HSD11B1 | Neutrophils | HPSE | TFH | SH3TC1 |
| DC | CCL13 | Neutrophils | SIGLEC5 | TFH | B3GAT1 |
| DC | CD209 | Neutrophils | VNN3 | TFH | KCNK5 |
| DC | CCL22 | Neutrophils | CRISPLD2 | TFH | THADA |
| DC | CCL17 | NK CD56bright cells | DUSP4 | TFH | SIRPG |
| DC | PPFIBP2 | NK CD56bright cells | DUSP4 | TFH | SLC7A10 |
| DC | NPR1 | NK CD56bright cells | PLA2G6 | TFH | KIAA1324 |
| Eosinophils | THBS1 | NK CD56bright cells | RRAD | TFH | MYO7A |
| Eosinophils | THBS1 | NK CD56bright cells | FOXJ1 | TFH | HEY1 |
| Eosinophils | SIAH1 | NK CD56bright cells | XCL1 | TFH | THADA |
| Eosinophils | TGIF1 | NK CD56bright cells | MPPED1 | TFH | ZNF764 |
| Eosinophils | HES1 | NK CD56bright cells | PLA2G6 | Tgd | FEZ1 |
| Eosinophils | HES1 | NK CD56bright cells | MUC3B | Tgd | C1orf61 |
| Eosinophils | KBTBD11 | NK CD56bright cells | LPCAT4 | Tgd | CD160 |
| Eosinophils | THBS4 | NK CD56bright cells | PLA2G6 | Tgd | TRDC |
| Eosinophils | KCNH2 | NK CD56bright cells | TRAPPC9 | Th1 cells | APOD |
| Eosinophils | ABHD2 | NK CD56bright cells | MADD | Th1 cells | BST2 |
| Eosinophils | RNASE2 | NK CD56dim cells | GTF3C1 | Th1 cells | DPP4 |
| Eosinophils | CLC | NK CD56dim cells | KIR2DL3 | Th1 cells | CCL4 |
| Eosinophils | PTGDR2 | NK CD56dim cells | KIR2DS5 | Th1 cells | SGCB |
| Eosinophils | EMR1 | NK CD56dim cells | GZMB | Th1 cells | LRP8 |
| Eosinophils | CD101 | NK CD56dim cells | KIR3DS1 | Th1 cells | BTG3 |
| Eosinophils | SYNJ1 | NK CD56dim cells | KIR2DS2 | Th1 cells | SYNGR3 |
| Eosinophils | CCR3 | NK CD56dim cells | KIR3DL1 | Th1 cells | CD38 |
| Eosinophils | HIST1H1C | NK CD56dim cells | TTC38 | Th1 cells | CD70 |
| Eosinophils | KCNH2 | NK CD56dim cells | KIR2DS4 | Th1 cells | DGKI |
| Eosinophils | IL5RA | NK CD56dim cells | KIR3DL3 | Th1 cells | LTA |
| Eosinophils | IL5RA | NK CD56dim cells | PMEPA1 | Th1 cells | IL12RB2 |
| Eosinophils | GALC | NK CD56dim cells | S1PR5 | Th1 cells | DUSP5 |
| Eosinophils | CAT | NK CD56dim cells | IL21R | Th1 cells | LRRN3 |
| Eosinophils | TIPARP | NK cells | IGFBP5 | Th1 cells | CSF2 |
| Eosinophils | SYNJ1 | NK cells | MAPRE3 | Th1 cells | IFNG |
| Eosinophils | TKTL1 | NK cells | ZNF747 | Th1 cells | CMAHP |
| Eosinophils | LRP5L | NK cells | XCL1 | Th1 cells | DPP4 |
| Eosinophils | RRP12 | NK cells | ZNF205 | Th1 cells | ATP9A |
| Eosinophils | TKTL1 | NK cells | TCTN2 | Th1 cells | APBB2 |
| Eosinophils | RCOR3 | NK cells | TRPV6 | Th1 cells | DOK5 |
| Eosinophils | SMPD3 | NK cells | FGF18 | Th1 cells | EGFL6 |
| Eosinophils | CYSLTR2 | NK cells | BCL2 | Th1 cells | ZBTB32 |
| Eosinophils | HRH4 | NK cells | TBXA2R | Th1 cells | IL22 |
| Eosinophils | ACACB | NK cells | NCR1 | Th1 cells | CTLA4 |
| Eosinophils | C9orf156 | NK cells | CDC5L | Th1 cells | HBEGF |
| Eosinophils | ACACB | NK cells | MRC2 | Th1 cells | APBB2 |
| Eosinophils | ACACB | NK cells | FZR1 | Th17 cells | IL17RA |
| Eosinophils | MYO15B | NK cells | ALDH1B1 | Th17 cells | RORC |
| iDC | BLVRB | NK cells | ADARB1 | Th17 cells | IL17A |
| iDC | TACSTD2 | NK cells | PSMD4 | Th17 cells | IL17A |
| iDC | CSF1R | NK cells | FUT5 | Th2 cells | DHFR |
| iDC | F13A1 | NK cells | FZR1 | Th2 cells | SNRPD1 |
| iDC | GSTT1 | NK cells | APBB2 | Th2 cells | GSTA4 |
| iDC | RAP1GAP | NK cells | PDLIM4 | Th2 cells | SMAD2 |
| iDC | VASH1 | NK cells | ZNF528 | Th2 cells | SMAD2 |
| iDC | FABP4 | NK cells | MCM3AP | Th2 cells | MB |
| iDC | PREP | NK cells | LDB3 | Th2 cells | CDC7 |
| iDC | MMP12 | NK cells | GNAS | Th2 cells | WDHD1 |
| iDC | CTNS | NK cells | NCR1 | Th2 cells | CDC25C |
| iDC | SYT17 | NK cells | NCR1 | Th2 cells | PMCH |
| iDC | CD1C | NK cells | TINAGL1 | Th2 cells | CXCR6 |
| iDC | GUCA1A | NK cells | PRX | Th2 cells | LAIR2 |
| iDC | CLEC10A | NK cells | SLC30A5 | Th2 cells | PTGIS |
| iDC | CD1B | NK cells | KANK2 | Th2 cells | ANK1 |
| iDC | CH25H | pDC | IL3RA | Th2 cells | CENPF |
| iDC | PPARG | T cells | ITM2A | Th2 cells | GATA3 |
| iDC | CD1E | T cells | LCK | Th2 cells | EVI5 |
| iDC | ABCG2 | T cells | LCK | Th2 cells | BIRC5 |
| iDC | FZD2 | T cells | CD3E | Th2 cells | PHEX |
| iDC | CD1A | T cells | SKAP1 | Th2 cells | CXCR6 |
| iDC | CD1E | T cells | CD2 | Th2 cells | SLC39A14 |
| iDC | SLC7A8 | T cells | CD28 | Th2 cells | MICAL2 |
| iDC | PDXK | T cells | CD96 | Th2 cells | ADCY1 |
| iDC | NUDT9 | T cells | CD3G | Th2 cells | LIMA1 |
| iDC | MS4A6A | T cells | TRAC | Th2 cells | NEIL3 |
| iDC | HS3ST2 | T cells | TRAC | Th2 cells | HELLS |
| iDC | DCSTAMP | T cells | PRKCQ | Th2 cells | AHI1 |
| iDC | LMAN2L | T cells | PRKCQ | Th2 cells | AHI1 |
| Macrophages | EMP1 | T cells | SH2D1A | Th2 cells | IL26 |
| Macrophages | SCARB2 | T cells | TRBC1 | Th2 cells | AHI1 |
| Macrophages | RAI14 | T cells | NCALD | Th2 cells | DHFR |
| Macrophages | CTSK | T cells | TRBC1 | TReg | FOXP3 |
| Macrophages | APOE | T cells | CD3D | TReg | FOXP3 |
| Macrophages | CD163 | T cells | YME1L1 | T cells | CD6 |
| Macrophages | FDX1 |  |  |  |  |

**Table S6 The immune cell signature 2 used in this work to estimate the relative abundance of immune cell infiltration.**

| **Cell_Type** | **Gene_Symbol** | | **Cell_Type** | **Gene_Symbol** |
| --- | --- | --- | --- | --- |
| Activated.B.cell | ADAM28 | Activated.dendritic.cell | | RHOA |
| Activated.B.cell | CD180 | Activated.dendritic.cell | | SLC25A37 |
| Activated.B.cell | CD79B | Activated.dendritic.cell | | TNFSF14 |
| Activated.B.cell | BLK | Activated.dendritic.cell | | TREML4 |
| Activated.B.cell | CD19 | Activated.dendritic.cell | | VNN2 |
| Activated.B.cell | MS4A1 | Activated.dendritic.cell | | XPO6 |
| Activated.B.cell | TNFRSF17 | Activated.dendritic.cell | | CLEC4C |
| Activated.B.cell | IGHM | Activated.dendritic.cell | | TNFAIP2 |
| Activated.B.cell | GNG7 | Activated.dendritic.cell | | UBD |
| Activated.B.cell | MICAL3 | Activated.dendritic.cell | | ACTR3 |
| Activated.B.cell | SPIB | Activated.dendritic.cell | | RAB1A |
| Activated.B.cell | HLA-DOB | Activated.dendritic.cell | | SLA |
| Activated.B.cell | IGKC | Activated.dendritic.cell | | HLA-DQA2 |
| Activated.B.cell | PNOC | Activated.dendritic.cell | | SIGLEC5 |
| Activated.B.cell | FCRL2 | Activated.dendritic.cell | | SLAMF9 |
| Activated.B.cell | BACH2 | CD56bright.natural.killer.cell | | ABAT |
| Activated.B.cell | CR2 | CD56bright.natural.killer.cell | | C11orf75 |
| Activated.B.cell | TCL1A | CD56bright.natural.killer.cell | | C5orf15 |
| Activated.B.cell | AKNA | CD56bright.natural.killer.cell | | CDHR1 |
| Activated.B.cell | ARHGAP25 | CD56bright.natural.killer.cell | | DCAF12 |
| Activated.B.cell | CCL21 | CD56bright.natural.killer.cell | | DYNLL1 |
| Activated.B.cell | CD27 | CD56bright.natural.killer.cell | | GPR137B |
| Activated.B.cell | CD38 | CD56bright.natural.killer.cell | | HCP5 |
| Activated.B.cell | CLEC17A | CD56bright.natural.killer.cell | | HDGFRP2 |
| Activated.B.cell | CLEC9A | CD56bright.natural.killer.cell | | KRT86 |
| Activated.B.cell | CLECL1 | CD56bright.natural.killer.cell | | MLST8 |
| Activated.CD4.T.cell | AIM2 | CD56bright.natural.killer.cell | | ELMOD3 |
| Activated.CD4.T.cell | BIRC3 | CD56bright.natural.killer.cell | | ENTPD5 |
| Activated.CD4.T.cell | BRIP1 | CD56bright.natural.killer.cell | | FAM119A |
| Activated.CD4.T.cell | CCL20 | CD56bright.natural.killer.cell | | FAM179A |
| Activated.CD4.T.cell | CCL4 | CD56bright.natural.killer.cell | | CLIC2 |
| Activated.CD4.T.cell | CCL5 | CD56bright.natural.killer.cell | | COX7A2L |
| Activated.CD4.T.cell | CCNB1 | CD56bright.natural.killer.cell | | CREB3L4 |
| Activated.CD4.T.cell | CCR7 | CD56bright.natural.killer.cell | | CSF1 |
| Activated.CD4.T.cell | DUSP2 | CD56bright.natural.killer.cell | | CSNK2A2 |
| Activated.CD4.T.cell | ESCO2 | CD56bright.natural.killer.cell | | CSTA |
| Activated.CD4.T.cell | ETS1 | CD56bright.natural.killer.cell | | CSTB |
| Activated.CD4.T.cell | EXO1 | CD56bright.natural.killer.cell | | CTPS |
| Activated.CD4.T.cell | EXOC6 | CD56bright.natural.killer.cell | | CTSD |
| Activated.CD4.T.cell | IARS | CD56bright.natural.killer.cell | | FST |
| Activated.CD4.T.cell | ITK | CD56bright.natural.killer.cell | | GATA2 |
| Activated.CD4.T.cell | KIF11 | CD56bright.natural.killer.cell | | GMPR |
| Activated.CD4.T.cell | KNTC1 | CD56bright.natural.killer.cell | | HDC |
| Activated.CD4.T.cell | NUF2 | CD56bright.natural.killer.cell | | HEY1 |
| Activated.CD4.T.cell | PRC1 | CD56bright.natural.killer.cell | | HOXA1 |
| Activated.CD4.T.cell | PSAT1 | CD56bright.natural.killer.cell | | HS2ST1 |
| Activated.CD4.T.cell | RGS1 | CD56bright.natural.killer.cell | | HS3ST1 |
| Activated.CD4.T.cell | RTKN2 | CD56bright.natural.killer.cell | | BCL11B |
| Activated.CD4.T.cell | SAMSN1 | CD56bright.natural.killer.cell | | CDH3 |
| Activated.CD4.T.cell | SELL | CD56bright.natural.killer.cell | | MYL6B |
| Activated.CD4.T.cell | TRAT1 | CD56bright.natural.killer.cell | | NAA16 |
| Activated.CD8.T.cell | ADRM1 | CD56bright.natural.killer.cell | | ClQA |
| Activated.CD8.T.cell | AHSA1 | CD56bright.natural.killer.cell | | ClQB |
| Activated.CD8.T.cell | C1GALT1C1 | CD56bright.natural.killer.cell | | CYP27B1 |
| Activated.CD8.T.cell | CCT6B | CD56bright.natural.killer.cell | | EIF3M |
| Activated.CD8.T.cell | CD37 | CD56dim.natural.killer.cell | | CYP27A1 |
| Activated.CD8.T.cell | CD3D | CD56dim.natural.killer.cell | | DDX55 |
| Activated.CD8.T.cell | CD3E | CD56dim.natural.killer.cell | | DYRK2 |
| Activated.CD8.T.cell | CD3G | CD56dim.natural.killer.cell | | RPL37A |
| Activated.CD8.T.cell | CD69 | CD56dim.natural.killer.cell | | NOTCH3 |
| Activated.CD8.T.cell | CD8A | CD56dim.natural.killer.cell | | AKR7A3 |
| Activated.CD8.T.cell | CETN3 | CD56dim.natural.killer.cell | | GPRC5C |
| Activated.CD8.T.cell | CSE1L | CD56dim.natural.killer.cell | | GRIN1 |
| Activated.CD8.T.cell | GEMIN6 | CD56dim.natural.killer.cell | | HLA-E |
| Activated.CD8.T.cell | GNLY | CD56dim.natural.killer.cell | | PORCN |
| Activated.CD8.T.cell | GPT2 | CD56dim.natural.killer.cell | | PSMC4 |
| Activated.CD8.T.cell | GZMA | CD56dim.natural.killer.cell | | UPP1 |
| Activated.CD8.T.cell | GZMH | CD56dim.natural.killer.cell | | IL21R |
| Activated.CD8.T.cell | GZMK | CD56dim.natural.killer.cell | | KIR2DS1 |
| Activated.CD8.T.cell | IL2RB | CD56dim.natural.killer.cell | | KIR2DS2 |
| Activated.CD8.T.cell | LCK | CD56dim.natural.killer.cell | | KIR2DS5 |
| Activated.CD8.T.cell | MPZL1 | Eosinophil | | GIPR |
| Activated.CD8.T.cell | NKG7 | Eosinophil | | KRT18P50 |
| Activated.CD8.T.cell | PIK3IP1 | Eosinophil | | LRMP |
| Activated.CD8.T.cell | PTRH2 | Eosinophil | | FOSB |
| Activated.CD8.T.cell | TIMM13 | Eosinophil | | RRP12 |
| Activated.CD8.T.cell | ZAP70 | Eosinophil | | GPR183 |
| Gamma.delta.T.cell | ACP5 | Eosinophil | | NR4A3 |
| Gamma.delta.T.cell | AQP9 | Eosinophil | | ST3GAL6 |
| Gamma.delta.T.cell | BTN3A2 | Eosinophil | | DEPDC5 |
| Gamma.delta.T.cell | C1orf54 | Eosinophil | | PDE6C |
| Gamma.delta.T.cell | CARD8 | Eosinophil | | PKD2L2 |
| Gamma.delta.T.cell | CCL18 | Eosinophil | | GPR65 |
| Gamma.delta.T.cell | CD209 | Eosinophil | | IL5RA |
| Gamma.delta.T.cell | CD33 | Eosinophil | | P2RY14 |
| Gamma.delta.T.cell | CD36 | Eosinophil | | DACH1 |
| Gamma.delta.T.cell | CDK5 | Eosinophil | | DAPK2 |
| Gamma.delta.T.cell | IL10RB | Eosinophil | | EMR3 |
| Gamma.delta.T.cell | KLRF1 | Immature.dendritic.cell | | ACADM |
| Gamma.delta.T.cell | LGALS1 | Immature.dendritic.cell | | AHCYL1 |
| Gamma.delta.T.cell | MAPK7 | Immature.dendritic.cell | | ALDH1A2 |
| Gamma.delta.T.cell | KLHL7 | Immature.dendritic.cell | | ALDH3A2 |
| Gamma.delta.T.cell | KRT80 | Immature.dendritic.cell | | ALDH9A1 |
| Gamma.delta.T.cell | LAMC1 | Immature.dendritic.cell | | ALOX15 |
| Gamma.delta.T.cell | LCORL | Immature.dendritic.cell | | AMT |
| Gamma.delta.T.cell | LMNB1 | Immature.dendritic.cell | | ARL1 |
| Gamma.delta.T.cell | MEIS3P1 | Immature.dendritic.cell | | ATIC |
| Gamma.delta.T.cell | MPL | Immature.dendritic.cell | | ATP5A1 |
| Gamma.delta.T.cell | FABP1 | Immature.dendritic.cell | | CAPZA1 |
| Gamma.delta.T.cell | FABP5 | Immature.dendritic.cell | | LILRA5 |
| Gamma.delta.T.cell | FADD | Immature.dendritic.cell | | RDX |
| Gamma.delta.T.cell | MFAP3L | Immature.dendritic.cell | | RRAGD |
| Gamma.delta.T.cell | MINPP1 | Immature.dendritic.cell | | TACSTD2 |
| Gamma.delta.T.cell | RPS24 | Immature.dendritic.cell | | INPP5F |
| Gamma.delta.T.cell | RPS7 | Immature.dendritic.cell | | RAB38 |
| Gamma.delta.T.cell | RPS9 | Immature.dendritic.cell | | PLAU |
| Gamma.delta.T.cell | DBNL | Immature.dendritic.cell | | CSF3R |
| Gamma.delta.T.cell | CCL13 | Immature.dendritic.cell | | SLC18A2 |
| Immature..B.cell | CD22 | Immature.dendritic.cell | | AMPD2 |
| Immature..B.cell | CYBB | Immature.dendritic.cell | | CLTB |
| Immature..B.cell | FAM129C | Immature.dendritic.cell | | C1orf162 |
| Immature..B.cell | FCRL1 | Macrophage | | AIF1 |
| Immature..B.cell | FCRL3 | Macrophage | | CCL1 |
| Immature..B.cell | FCRL5 | Macrophage | | CCL14 |
| Immature..B.cell | FCRLA | Macrophage | | CCL23 |
| Immature..B.cell | HDAC9 | Macrophage | | CCL26 |
| Immature..B.cell | HLA-DQA1 | Macrophage | | CD300LB |
| Immature..B.cell | HVCN1 | Macrophage | | CNR1 |
| Immature..B.cell | KIAA0226 | Macrophage | | CNR2 |
| Immature..B.cell | NCF1 | Macrophage | | EIF1 |
| Immature..B.cell | NCF1B | Macrophage | | EIF4A1 |
| Immature..B.cell | P2RY10 | Macrophage | | FPR1 |
| Immature..B.cell | SP100 | Macrophage | | FPR2 |
| Immature..B.cell | TXNIP | Macrophage | | FRAT2 |
| Immature..B.cell | STAP1 | Macrophage | | GPR27 |
| Immature..B.cell | TAGAP | Macrophage | | GPR77 |
| Immature..B.cell | ZCCHC2 | Macrophage | | RNASE2 |
| Regulatory.T.cell | CCL3L1 | Macrophage | | MS4A2 |
| Regulatory.T.cell | CD72 | Macrophage | | BASP1 |
| Regulatory.T.cell | CLEC5A | Macrophage | | IGSF6 |
| Regulatory.T.cell | FOXP3 | Macrophage | | HK3 |
| Regulatory.T.cell | ITGA4 | Macrophage | | VNN1 |
| Regulatory.T.cell | L1CAM | Macrophage | | FES |
| Regulatory.T.cell | LIPA | Macrophage | | NPL |
| Regulatory.T.cell | LRP1 | Macrophage | | FZD2 |
| Regulatory.T.cell | LRRC42 | Macrophage | | FAM198B |
| Regulatory.T.cell | MARCO | Macrophage | | HNMT |
| Regulatory.T.cell | MMP12 | Macrophage | | SLC15A3 |
| Regulatory.T.cell | MNDA | Macrophage | | CD4 |
| Regulatory.T.cell | MRC1 | Macrophage | | TXNDC3 |
| Regulatory.T.cell | MS4A6A | Macrophage | | FRMD4A |
| Regulatory.T.cell | PELO | Macrophage | | CRYBB1 |
| Regulatory.T.cell | PLEK | Macrophage | | HRH1 |
| Regulatory.T.cell | PRSS23 | Macrophage | | WNT5B |
| Regulatory.T.cell | PTGIR | Mast.cell | | ADAMTS3 |
| Regulatory.T.cell | ST8SIA4 | Mast.cell | | CPA3 |
| Regulatory.T.cell | STAB1 | Mast.cell | | CMA1 |
| T.follicular.helper.cell | B3GAT1 | Mast.cell | | CTSG |
| T.follicular.helper.cell | CDK5R1 | Mast.cell | | ARHGAP15 |
| T.follicular.helper.cell | PDCD1 | Mast.cell | | CPM |
| T.follicular.helper.cell | BCL6 | Mast.cell | | FCN1 |
| T.follicular.helper.cell | CD200 | Mast.cell | | FTL |
| T.follicular.helper.cell | CD83 | Mast.cell | | HSPA6 |
| T.follicular.helper.cell | CD84 | Mast.cell | | ITGA9 |
| T.follicular.helper.cell | FGF2 | Mast.cell | | RNASE3 |
| T.follicular.helper.cell | GPR18 | Mast.cell | | S100A4 |
| T.follicular.helper.cell | CEBPA | Mast.cell | | SIGLEC8 |
| T.follicular.helper.cell | CECR1 | Mast.cell | | SLC6A4 |
| T.follicular.helper.cell | CLEC10A | Mast.cell | | PTGS2 |
| T.follicular.helper.cell | CLEC4A | Mast.cell | | EGR3 |
| T.follicular.helper.cell | CSF1R | Mast.cell | | PILRA |
| T.follicular.helper.cell | CTSS | MDSC | | CCR2 |
| T.follicular.helper.cell | DMN | MDSC | | CD14 |
| T.follicular.helper.cell | DPP4 | MDSC | | CD2 |
| T.follicular.helper.cell | LRRC32 | MDSC | | CD86 |
| T.follicular.helper.cell | MC5R | MDSC | | CXCR4 |
| T.follicular.helper.cell | MICA | MDSC | | FCGR2A |
| T.follicular.helper.cell | NCAM1 | MDSC | | FCGR2B |
| T.follicular.helper.cell | NCR2 | MDSC | | FCGR3A |
| T.follicular.helper.cell | NRP1 | MDSC | | FERMT3 |
| T.follicular.helper.cell | PDCD1LG2 | MDSC | | GPSM3 |
| T.follicular.helper.cell | PDCD6 | MDSC | | IL18BP |
| T.follicular.helper.cell | PRDX1 | MDSC | | IL4R |
| T.follicular.helper.cell | RAE1 | MDSC | | ITGAL |
| T.follicular.helper.cell | RAET1E | MDSC | | ITGAM |
| T.follicular.helper.cell | SIGLEC7 | MDSC | | PARVG |
| T.follicular.helper.cell | SIGLEC9 | MDSC | | PSAP |
| T.follicular.helper.cell | TYRO3 | MDSC | | PTGER2 |
| T.follicular.helper.cell | CHST12 | MDSC | | PTGES2 |
| T.follicular.helper.cell | CLIC3 | MDSC | | S100A8 |
| T.follicular.helper.cell | IVNS1ABP | MDSC | | S100A9 |
| T.follicular.helper.cell | KIR2DL2 | Monocyte | | ASGR2 |
| T.follicular.helper.cell | LGMN | Monocyte | | CFP |
| Type.1.T.helper.cell | CD70 | Monocyte | | ASGR1 |
| Type.1.T.helper.cell | TBX21 | Monocyte | | CD1D |
| Type.1.T.helper.cell | ADAM8 | Monocyte | | UPK3A |
| Type.1.T.helper.cell | AHCYL2 | Monocyte | | ACTG1 |
| Type.1.T.helper.cell | ALCAM | Monocyte | | ANXA5 |
| Type.1.T.helper.cell | B3GALNT1 | Monocyte | | ATP6V1B2 |
| Type.1.T.helper.cell | BBS12 | Monocyte | | CFL1 |
| Type.1.T.helper.cell | BST1 | Monocyte | | DAZAP2 |
| Type.1.T.helper.cell | CD151 | Monocyte | | CTBS |
| Type.1.T.helper.cell | CD47 | Monocyte | | EMR4P |
| Type.1.T.helper.cell | CD48 | Monocyte | | HIVEP2 |
| Type.1.T.helper.cell | CD52 | Monocyte | | MARCKSL1 |
| Type.1.T.helper.cell | CD53 | Monocyte | | MBP |
| Type.1.T.helper.cell | CD59 | Monocyte | | MMP15 |
| Type.1.T.helper.cell | CD6 | Monocyte | | PNPLA6 |
| Type.1.T.helper.cell | CD68 | Monocyte | | TMBIM6 |
| Type.1.T.helper.cell | CD7 | Monocyte | | PQBP1 |
| Type.1.T.helper.cell | CD96 | Monocyte | | TEX264 |
| Type.1.T.helper.cell | CFHR3 | Monocyte | | IKZF1 |
| Type.1.T.helper.cell | CHRM3 | Natural.killer.cell | | AKT3 |
| Type.1.T.helper.cell | CLEC7A | Natural.killer.cell | | AXL |
| Type.1.T.helper.cell | COL23A1 | Natural.killer.cell | | BST2 |
| Type.1.T.helper.cell | COL4A4 | Natural.killer.cell | | CDH2 |
| Type.1.T.helper.cell | COL5A3 | Natural.killer.cell | | CRTAM |
| Type.1.T.helper.cell | DAB1 | Natural.killer.cell | | CSF2RA |
| Type.1.T.helper.cell | DLEU7 | Natural.killer.cell | | CTSZ |
| Type.1.T.helper.cell | DOC2B | Natural.killer.cell | | CXCL1 |
| Type.1.T.helper.cell | EMP1 | Natural.killer.cell | | CYTH1 |
| Type.1.T.helper.cell | F12 | Natural.killer.cell | | DAXX |
| Type.1.T.helper.cell | FURIN | Natural.killer.cell | | DGKH |
| Type.1.T.helper.cell | GAB3 | Natural.killer.cell | | DLL4 |
| Type.1.T.helper.cell | GATM | Natural.killer.cell | | DPYD |
| Type.1.T.helper.cell | GFPT2 | Natural.killer.cell | | ERBB3 |
| Type.1.T.helper.cell | GPR25 | Natural.killer.cell | | F11R |
| Type.1.T.helper.cell | GREM2 | Natural.killer.cell | | FAM27A |
| Type.1.T.helper.cell | HAVCR1 | Natural.killer.cell | | FAM49A |
| Type.1.T.helper.cell | HSD11B1 | Natural.killer.cell | | FASLG |
| Type.1.T.helper.cell | HUNK | Natural.killer.cell | | FCGR1A |
| Type.1.T.helper.cell | IGF2 | Natural.killer.cell | | FN1 |
| Type.1.T.helper.cell | RCSD1 | Natural.killer.cell | | FSTL1 |
| Type.1.T.helper.cell | RYR1 | Natural.killer.cell | | FUCA1 |
| Type.1.T.helper.cell | SAV1 | Natural.killer.cell | | GBP3 |
| Type.1.T.helper.cell | SELE | Natural.killer.cell | | GLS2 |
| Type.1.T.helper.cell | SELP | Natural.killer.cell | | GRB2 |
| Type.1.T.helper.cell | SH3KBP1 | Natural.killer.cell | | LST1 |
| Type.1.T.helper.cell | SIT1 | Natural.killer.cell | | BCL2 |
| Type.1.T.helper.cell | SLC35B3 | Natural.killer.cell | | CDC5L |
| Type.1.T.helper.cell | SIGLEC10 | Natural.killer.cell | | FGF18 |
| Type.1.T.helper.cell | SKAP1 | Natural.killer.cell | | FUT5 |
| Type.1.T.helper.cell | THUMPD2 | Natural.killer.cell | | FZR1 |
| Type.1.T.helper.cell | TIGIT | Natural.killer.cell | | GAGE2 |
| Type.1.T.helper.cell | ZEB2 | Natural.killer.cell | | IGFBP5 |
| Type.1.T.helper.cell | ENC1 | Natural.killer.cell | | KANK2 |
| Type.1.T.helper.cell | FAM134B | Natural.killer.cell | | LDB3 |
| Type.1.T.helper.cell | FBXO30 | Natural.killer.T.cell | | BTN2A2 |
| Type.1.T.helper.cell | FCGR2C | Natural.killer.T.cell | | CD101 |
| Type.1.T.helper.cell | STAC | Natural.killer.T.cell | | CD109 |
| Type.1.T.helper.cell | LTC4S | Natural.killer.T.cell | | CNPY3 |
| Type.1.T.helper.cell | MAN1B1 | Natural.killer.T.cell | | CNPY4 |
| Type.1.T.helper.cell | MDH1 | Natural.killer.T.cell | | CREB1 |
| Type.1.T.helper.cell | MMD | Natural.killer.T.cell | | CRTC2 |
| Type.1.T.helper.cell | RGS16 | Natural.killer.T.cell | | CRTC3 |
| Type.1.T.helper.cell | IL12A | Natural.killer.T.cell | | CSF2 |
| Type.1.T.helper.cell | P2RX5 | Natural.killer.T.cell | | KLRC1 |
| Type.1.T.helper.cell | CD97 | Natural.killer.T.cell | | FUT4 |
| Type.1.T.helper.cell | ITGB4 | Natural.killer.T.cell | | ICAM2 |
| Type.1.T.helper.cell | ICAM3 | Natural.killer.T.cell | | IL32 |
| Type.1.T.helper.cell | METRNL | Natural.killer.T.cell | | LAMP2 |
| Type.1.T.helper.cell | TNFRSF1A | Natural.killer.T.cell | | LILRB5 |
| Type.1.T.helper.cell | IRF1 | Natural.killer.T.cell | | KLRG1 |
| Type.1.T.helper.cell | HTR2B | Natural.killer.T.cell | | HSPA4 |
| Type.1.T.helper.cell | CALD1 | Natural.killer.T.cell | | HSPB6 |
| Type.1.T.helper.cell | MOCOS | Natural.killer.T.cell | | ISM2 |
| Type.1.T.helper.cell | TRAF3IP2 | Natural.killer.T.cell | | ITIH2 |
| Type.1.T.helper.cell | TLR8 | Natural.killer.T.cell | | KDM4C |
| Type.1.T.helper.cell | TRAF1 | Natural.killer.T.cell | | KIR2DS4 |
| Type.1.T.helper.cell | DUSP14 | Natural.killer.T.cell | | KIRREL3 |
| Type.17.T.helper.cell | IL17A | Natural.killer.T.cell | | SDCBP |
| Type.17.T.helper.cell | IL17RA | Natural.killer.T.cell | | NFATC2IP |
| Type.17.T.helper.cell | C2CD4A | Natural.killer.T.cell | | MICB |
| Type.17.T.helper.cell | C2CD4B | Natural.killer.T.cell | | KIR2DL1 |
| Type.17.T.helper.cell | CA2 | Natural.killer.T.cell | | KIR2DL3 |
| Type.17.T.helper.cell | CCDC65 | Natural.killer.T.cell | | KIR3DL1 |
| Type.17.T.helper.cell | CEACAM3 | Natural.killer.T.cell | | KIR3DL2 |
| Type.17.T.helper.cell | IL17C | Natural.killer.T.cell | | NCR1 |
| Type.17.T.helper.cell | IL17F | Natural.killer.T.cell | | FOSL1 |
| Type.17.T.helper.cell | IL17RC | Natural.killer.T.cell | | TSLP |
| Type.17.T.helper.cell | IL17RE | Natural.killer.T.cell | | SLC7A7 |
| Type.17.T.helper.cell | IL23A | Natural.killer.T.cell | | SPP1 |
| Type.17.T.helper.cell | ILDR1 | Natural.killer.T.cell | | TREM2 |
| Type.17.T.helper.cell | LONRF3 | Natural.killer.T.cell | | UBASH3A |
| Type.17.T.helper.cell | SH2D6 | Natural.killer.T.cell | | YBX2 |
| Type.17.T.helper.cell | TNIP2 | Natural.killer.T.cell | | CCDC88A |
| Type.17.T.helper.cell | ABCA1 | Natural.killer.T.cell | | CLEC1A |
| Type.17.T.helper.cell | ABCB1 | Natural.killer.T.cell | | THBD |
| Type.17.T.helper.cell | ADAMTS12 | Natural.killer.T.cell | | PDPN |
| Type.17.T.helper.cell | ANK1 | Natural.killer.T.cell | | VCAM1 |
| Type.17.T.helper.cell | ANKRD22 | Natural.killer.T.cell | | EMR1 |
| Type.17.T.helper.cell | B3GALT2 | Neutrophil | | CREB5 |
| Type.17.T.helper.cell | CAMTA1 | Neutrophil | | CDA |
| Type.17.T.helper.cell | CCR9 | Neutrophil | | CHST15 |
| Type.17.T.helper.cell | CD40 | Neutrophil | | S100A12 |
| Type.17.T.helper.cell | GPR44 | Neutrophil | | APOBEC3A |
| Type.17.T.helper.cell | IFT80 | Neutrophil | | CASP5 |
| Type.2.T.helper.cell | ASB2 | Neutrophil | | MMP25 |
| Type.2.T.helper.cell | CSRP2 | Neutrophil | | HAL |
| Type.2.T.helper.cell | DAPK1 | Neutrophil | | C1orf183 |
| Type.2.T.helper.cell | DLC1 | Neutrophil | | FFAR2 |
| Type.2.T.helper.cell | DNAJC12 | Neutrophil | | MAK |
| Type.2.T.helper.cell | DUSP6 | Neutrophil | | CXCR1 |
| Type.2.T.helper.cell | GNAI1 | Neutrophil | | STEAP4 |
| Type.2.T.helper.cell | LAMP3 | Neutrophil | | MGAM |
| Type.2.T.helper.cell | NRP2 | Neutrophil | | BTNL8 |
| Type.2.T.helper.cell | OSBPL1A | Neutrophil | | CXCR2 |
| Type.2.T.helper.cell | PDE4B | Neutrophil | | TNFRSF10C |
| Type.2.T.helper.cell | PHLDA1 | Neutrophil | | VNN3 |
| Type.2.T.helper.cell | PLA2G4A | Plasmacytoid.dendritic.cell | | CBX6 |
| Type.2.T.helper.cell | RAB27B | Plasmacytoid.dendritic.cell | | DAB2 |
| Type.2.T.helper.cell | RBMS3 | Plasmacytoid.dendritic.cell | | DDX17 |
| Type.2.T.helper.cell | RNF125 | Plasmacytoid.dendritic.cell | | HIGD1A |
| Type.2.T.helper.cell | TMPRSS3 | Plasmacytoid.dendritic.cell | | IDH3A |
| Type.2.T.helper.cell | GATA3 | Plasmacytoid.dendritic.cell | | IL3RA |
| Type.2.T.helper.cell | BIRC5 | Plasmacytoid.dendritic.cell | | MAGED1 |
| Type.2.T.helper.cell | CDC25C | Plasmacytoid.dendritic.cell | | NUCB2 |
| Type.2.T.helper.cell | CDC7 | Plasmacytoid.dendritic.cell | | OFD1 |
| Type.2.T.helper.cell | CENPF | Plasmacytoid.dendritic.cell | | OGT |
| Type.2.T.helper.cell | CXCR6 | Plasmacytoid.dendritic.cell | | PDIA4 |
| Type.2.T.helper.cell | DHFR | Plasmacytoid.dendritic.cell | | SERTAD2 |
| Type.2.T.helper.cell | EVI5 | Plasmacytoid.dendritic.cell | | SIRPA |
| Type.2.T.helper.cell | GSTA4 | Plasmacytoid.dendritic.cell | | TMED2 |
| Type.2.T.helper.cell | HELLS | Plasmacytoid.dendritic.cell | | ENG |
| Type.2.T.helper.cell | IL26 | Plasmacytoid.dendritic.cell | | FCAR |
| Type.2.T.helper.cell | LAIR2 | Plasmacytoid.dendritic.cell | | IGF1 |
| Activated.dendritic.cell | ABCD1 | Plasmacytoid.dendritic.cell | | ITGA2B |
| Activated.dendritic.cell | C1QC | Plasmacytoid.dendritic.cell | | GABARAP |
| Activated.dendritic.cell | CAPG | Plasmacytoid.dendritic.cell | | GPX1 |
| Activated.dendritic.cell | CCL3L3 | Plasmacytoid.dendritic.cell | | KRT23 |
| Activated.dendritic.cell | CD207 | Plasmacytoid.dendritic.cell | | PROK2 |
| Activated.dendritic.cell | CD302 | Plasmacytoid.dendritic.cell | | RALB |
| Activated.dendritic.cell | ATP5B | Plasmacytoid.dendritic.cell | | RETNLB |
| Activated.dendritic.cell | ATP5L | Plasmacytoid.dendritic.cell | | RNF141 |
| Activated.dendritic.cell | ATP6V1A | Plasmacytoid.dendritic.cell | | SEC14L1 |
| Activated.dendritic.cell | BCL2L1 | Plasmacytoid.dendritic.cell | | SEPX1 |
| Activated.dendritic.cell | C1QB | Plasmacytoid.dendritic.cell | | EMP3 |
| Activated.dendritic.cell | SNURF | Plasmacytoid.dendritic.cell | | CD300LF |
| Activated.dendritic.cell | SPCS3 | Plasmacytoid.dendritic.cell | | ABTB1 |
| Activated.dendritic.cell | CCNA1 | Plasmacytoid.dendritic.cell | | KLHL21 |
| Activated.dendritic.cell | CEACAM8 | Plasmacytoid.dendritic.cell | | PHRF1 |
| Activated.dendritic.cell | NOS2 | Activated.dendritic.cell | | TREM1 |
| Activated.dendritic.cell | SRA1 | Activated.dendritic.cell | | TREML1 |
| Activated.dendritic.cell | TNFRSF6B |  | |  |

**Table S7 The list of ferroptosis regulators to calculate FPI.**

| **Symbol** | **Type** |
| --- | --- |
| LPCAT3 | Positive |
| ACSL4 | Positive |
| NCOA4 | Positive |
| ALOX15 | Positive |
| GPX4 | Positive |
| SLC3A2 | Positive |
| SLC7A11 | Positive |
| NFE2L2 | Positive |
| NOX1 | Positive |
| NOX3 | Positive |
| NOX4 | Positive |
| NOX5 | Positive |
| FDFT1 | Negative |
| HMGCR | Negative |
| COQ10A | Negative |
| COQ10B | Negative |

**Table S8 The list of MDH gene signature in this study.**

| **DEGs** | **DEGs** | **DEGs** |
| --- | --- | --- |
| ITGA1 | CPEB4 | ALYREF |
| ADH4 | FRK | FAM43B |
| ZC3H6 | PPARGC1A | C1orf116 |
| IRS2 | LRP2BP | MFSD5 |
| SLCO4C1 | SEPSECS | VWA1 |
| CREBRF | FAM20A | ZNF114 |
| PPM1L | RASD1 | PHYHD1 |
| AK9 | RUNDC3B | DAG1 |
| L3MBTL4 | FGL1 | ZNF584 |
| SORBS2 | MYOM1 | CCDC8 |
| CLGN | SIX4 | CHST14 |
| NR3C2 | CDC7 | SNRNP25 |
| ADD3 | GCKR | CHAF1B |
| CYP4V2 | TUBE1 | ENDOD1 |
| GALNT13 | RORA | SLC37A2 |
| AMDHD1 | ACSL4 | FOXJ1 |
| INHBE | SYNE2 | PAX8 |
| LOXL4 | AGA | BCAS4 |
| KANK4 | ACSM3 | ASIC1 |
| BICC1 | CPTP | P3H3 |
| TNFSF4 | ARHGEF35 | FSCN1 |
| LEPR | MT1M | USP2 |
| ATAD3A | MT1A | ZBED6CL |
| BCL9L |  |  |

DEGs: differentially expressed genes.
